# Deep-broad learning network model for precision identification and diagnosis of grape leaf diseases

**DOI:** 10.3389/fpls.2025.1611301

**Published:** 2025-09-10

**Authors:** Weimin Zhang, Yangyang Liu, Ya Feng, Longzhe Quan, Guoxiang Zhang, Chunyu Zhang

**Affiliations:** ^1^ School of Mechanical Engineering, Anhui University of Technology, Ma’anshan, China; ^2^ College of Intelligent Manufacturing, Anhui Science and Technology University, Chuzhou, China; ^3^ School of Engineering, Anhui Agricultural University, Hefei, China; ^4^ College of Information Engineering, Shaoxing Vocational & Technical College, Shaoxing, China

**Keywords:** grape leaf diseases, disease recognition, broad learning, lesion segmentation, deep learning, diseases diagnosis

## Abstract

This paper addresses the problem of rapid, precise, and efficient identification and diagnosis of grape leaf diseases by proposing the Deep-Broad Learning Network Model (ABLSS), which combines a Broad Learning network model with deep learning techniques. The model is optimized using the Adam algorithm based on BLS, and incorporates the LTM mechanism, which significantly enhances learning efficiency, stability, and recognition accuracy. Additionally, by integrating deep learning network optimization techniques, a SENet attention mechanism is added between the mapping and enhancement layers of BLS. Furthermore, based on the U-Net segmentation model, the method integrates dilated spatial pyramid pooling and feature pyramid networks. Dilated convolutions with varying dilation rates are used to capture multi-scale contextual information, which providing rich semantic information and high-resolution details during the decoding process. This improves the ABLSS model’s ability to identify small disease spots. Experimental results show that the ABLSS model achieves the highest recognition accuracy for three types of diseases with similar features on grape leaves, with an average accuracy improvement of 7.69% over BLS and 4.48% over deep learning networks. The MIOU of the segmentation model reaches 86.61%, which is a 6.48% improvement over the original U-Net model, and the MPA is 90.23%, a 8.09% improvement over the original U-Net. These results demonstrate that the proposed method significantly improves the algorithm’s recognition accuracy for small and irregular complex images. The ABLSS model recognizes images 0.375 seconds faster than the deep learning network, achieving a 72.12% speed improvement, thereby significantly enhancing the recognition efficiency of fine features. The ABLSS model combines the high recognition accuracy of deep learning with the fast processing speed of Broad Learning, while overcoming the limitations of BLS in recognizing complex images. This study provides valuable support for the development of smart orchard technologies and the optimization of learning network models.

## 1 Introduction

Grapes play an important role in agricultural economics, but their leaves are highly susceptible to pathogen attacks, leading to an annual yield loss of more than 30%, which severely impacts grape quality and yield, hindering the healthy development of the grape industry (Allaby, 2023; Ashley, 2024; Zhang et al., 2024). The condition of grape leaves directly reflects the development of the fruit; therefore, the rapid and precise identification of leaf diseases is one of the key directions in smart orchard research. However, the complex natural environment significantly impacts the extraction and identification of leaf diseases.

With the development of intelligent learning networks, deep learning models have made preliminary achievements in recognizing crop diseases and pests (Yuan et al., 2022; Merchant et al., 2023; Mu et al., 2024). Song et al. (2024) developed the MS-ppy-PDMS e-skin system, which captures underwater communication commands through a multilayer perceptron (MLP) neural network, enabling rapid and accurate analysis and identification. (Liu et al., 2024), Wang et al. (2024) proposed a deep learning algorithm for colorimetric micro-needle sensors (CMS), achieving visual recognition and evaluation of meat freshness. The CMS system utilizes anthocyanin responses to pH changes in structure and color to achieve *in-situ* visualization of meat freshness. Arabinda et al. (2023) proposed a new classification model that improves the performance of corn leaf disease recognition by fine-tuning a support vector machine (SVM) using DenseNet201 deep features and Bayesian optimization techniques. Li et al. (2024) researched a new GhostNet_Triplet_YOLOv8s algorithm, which integrates the lightweight GhostNet structure into YOLO v8s to effectively improve the speed and accuracy of disease recognition. Ashwini and Sellam (2024) proposed a hybrid model that combines Whale Optimization Algorithm and Joint Search Mechanisms to optimize a hybrid 3DCNN-RNN model, strengthening 3D images for corn leaf disease recognition using four layers of MaxPooling3D and six 3D convolutional layers. Sharma et al. (Vivek et al., 2024) developed a new model called ClGan, which reduces the number of parameters by combining with deep CNN, effectively solving issues such as background blur and data imbalance in field-collected datasets. These studies all achieve leaf disease recognition, but they are focused on field crops, and the conditions of field environments differ from those of orchard environments, making them unsuitable for grape leaf disease recognition.

Pan et al. (2024) conducted research on grape rootstock leaf recognition based on deep learning, constructing a dataset containing 13,547 images and using convolutional neural networks (CNNs) such as GoogleNet, ResNet-50, ResNet-101, and VGG-16 for automatic recognition, improving recognition accuracy. Mahmud and Hong (2022) developed a CNN-based semantic segmentation framework applicable to plant structures. This study employs a CNN architecture-based U-NET technique combined with the deep convolutional neural network VGG16 to construct an optimization function targeting microstructural characteristics. By utilizing a permutation operator to generate multiclass feature maps from stacked data, Improved accuracy. Prasad et al. (2024) used a deep convolutional neural network (DCNN) classifier for multi-class grape leaf disease recognition, enhancing performance and generalization by adding auxiliary layers and using public datasets for classification. Gürkan et al. (2024) utilized ESRGAN data augmentation and GASVM feature selection techniques to prevent overfitting and used ESRGAN to create synthetic images for obtaining detailed texture information, achieving automatic identification of grape leaf types while reducing human error and workload. Mahmud et al. (2024) proposes a lightweight deep learning-based method for automatic classification of mango leaf diseases. The model incorporates customized layers into the DenseNet architecture, enabling generalized recognition across diverse mango leaf datasets. With significantly accelerated training processes, this model presents an effective diagnostic solution for mango leaf diseases. These studies are all based on deep learning techniques for plant disease recognition. While they achieve disease recognition, deep learning requires high-quality and large datasets, performs poorly on small samples, and lacks generalization in complex environments. It also has high computational resource demands, and the plasticity loss in deep continual learning leads to a loss of learning ability when switching between tasks (Dohare et al., 2024).

To address the issues of long training times and low efficiency in deep learning models, Cao et al. (Heling et al., 2023) proposed a Broad Learning System (BLS), which enhances the discriminative ability of BLS for data features by introducing local sensitive discriminant analysis, improving image classification performance. Jin et al. (Junwei et al., 2023) proposed a BLS model based on flexible labels, improving classification performance by relaxing label boundaries and enhancing intra-label diversity. Liu et al. (Licheng et al., 2023) proposed a BLS based on modal regression, which optimizes output weights by replacing the least mean square error criterion, enhancing robustness to noise and outlier data. These studies demonstrate the feasibility and stability of using Broad Learning for recognition. Fu et al. (Rongrong et al., 2024). proposed a Transformer-BLS based on a multi-head attention mechanism and incremental learning algorithms, achieving incremental learning for feature nodes, enhancement nodes, input data, and sub-BLS layers, with performance verified on multiple image classification datasets. Muhammad et al. (2023) proposed a CNN-based BLS for recognizing threat objects in security X-ray scans, optimizing classification performance without manual intervention to adjust the BLS architecture. These studies combine Broad Learning with deep learning to form recognition algorithms that complement each other. However, the aforementioned studies are mainly focused on industrial applications and have not addressed agricultural leaf disease recognition and classification. The agricultural environment is complex and influenced by factors such as weather, lighting, occlusion, and shaking, making these studies unsuitable for agricultural applications.

Therefore, to address the problem of grape leaf disease identification with similar color characteristics and irregular distribution of disease spots, this paper carries out the fusion-optimization research of broad learning and deep learning, and proposes the ABLSS Deep-Broad Learning Network Model of grape disease identification and diagnosis with complementary advantages of two algorithms. The model also integrates dilated spatial pyramid pooling and feature pyramid networks, and uses dilated convolution with varying dilation rates are used to capture multi-scale contextual information, which further improves the algorithm ‘s ability to analyze and extract subtle features of complex images, and improves the ABLSS model ‘s ability to identify small disease spots. This paper can accurately, quickly and efficiently identify grape diseases in complex natural environment, measure the proportion of disease spots and diagnose the disease, provide support for disease diagnosis and accurate treatment, realize efficient management of vineyards, and promote the development of intelligent orchards.

## 2 Materials and methods

### 2.1 Grape leaf disease dataset processing

This study uses the image samples from the official open-source dataset *Plant Village* in the AI Challenger Global Challenge Plant Disease Classification project (Grape leaf disease, 2022). Three common and high-incidence grape diseases in vineyards are selected for research, namely Grape Black Rot, Grape Black Measles, and Grape Leaf Blight, as shown in

**Figure 1:**
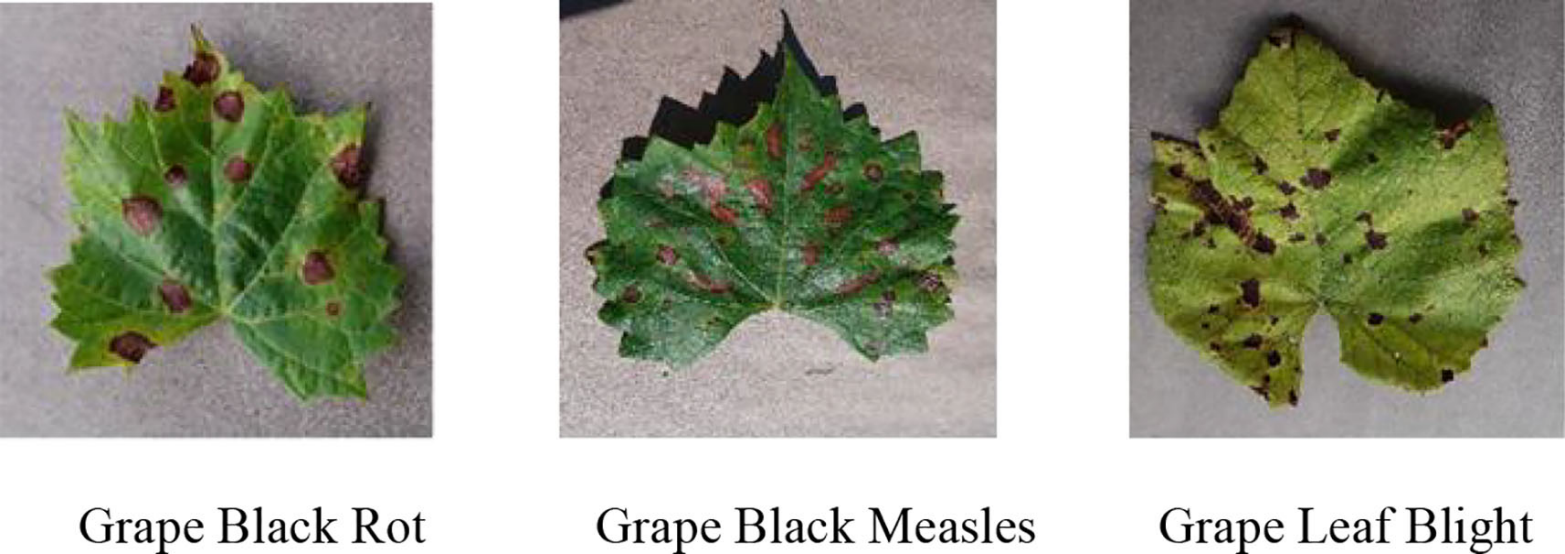
Three grape leaf diseases.

As shown in **Figure 1**, The lesion colors of the three diseases are dark brown, yellowish-brown, and grayish-brown, respectively. Their lesion shapes are irregular circular, fan-shaped scorch, and long spindle-shaped or elliptical. The color and shape of the lesions caused by the three diseases are highly similar. Therefore, color segmentation cannot be directly applied for recognition, significantly increasing the difficulty of recognition and differentiation. This paper performs a comprehensive analysis and evaluation based on lesion shape details, edge features, and lesion distribution patterns. It further optimizes the BLS by integrating deep learning methods to achieve precise disease recognition.

In this study, the Labelme annotation tool was used to annotate the images in the dataset in detail, accurately identifying and marking the leaf edges as well as the characteristic edges of various diseases such as Grape Black Rot, Grape Black Measles, and Grape Leaf Blight. The dataset is shown in **Table 1**.

**Table 1:** Grape leaf disease dataset.

Dataset	Sample size	Training set	Test set
Grape Black Rot	1884	1319	565
Grape Black Measles	1923	1346	577
Grape Leaf Blight	1789	1252	537
Aggregate	5596	3917	1679

**Figure 2:**
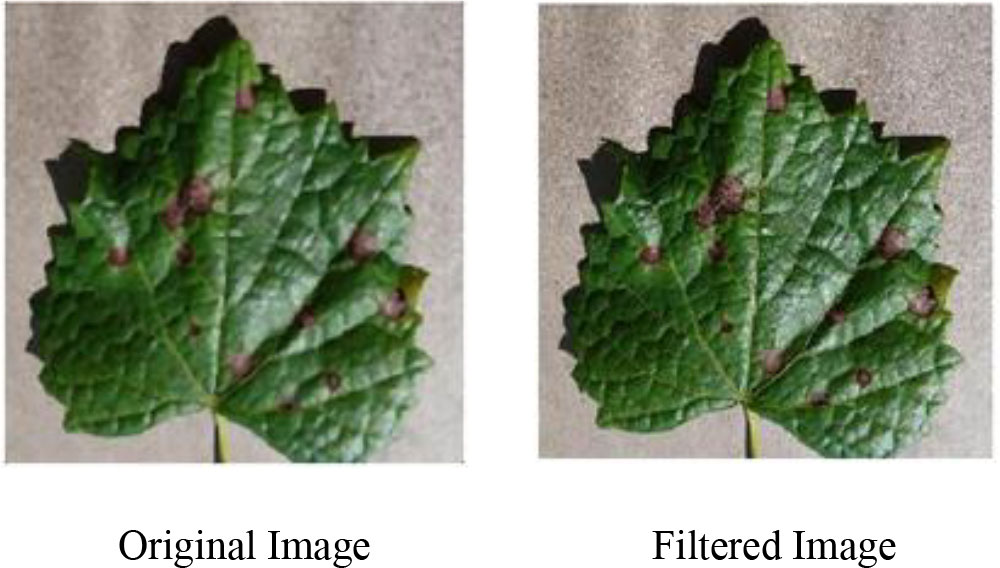
Comparison of median filtering denoising image.


To reduce background noise interference in the experimental images, a median filtering algorithm is used for image preprocessing to enhance the key features in the images. The algorithm traverses each pixel point using a sliding window, then sorts the grayscale values of all pixels and selects the middle value as the new grayscale value for the current pixel. This method removes randomly occurring isolated noise points and ensures that the pixel values in the image reflect the real situation. The filtering method is shown in Equation 1. A comparison of the images before and after denoising using the median filter is shown in **Figure 2**.

(1) y(n)=med[x(i−N),…,x(i),…,x(i+N)]

*y*(*n*)-the output value of the median filter; *med*(.)-the median function; *x*(*i*)-the pixel value of the center point of the sliding window.

**Figure 3:**
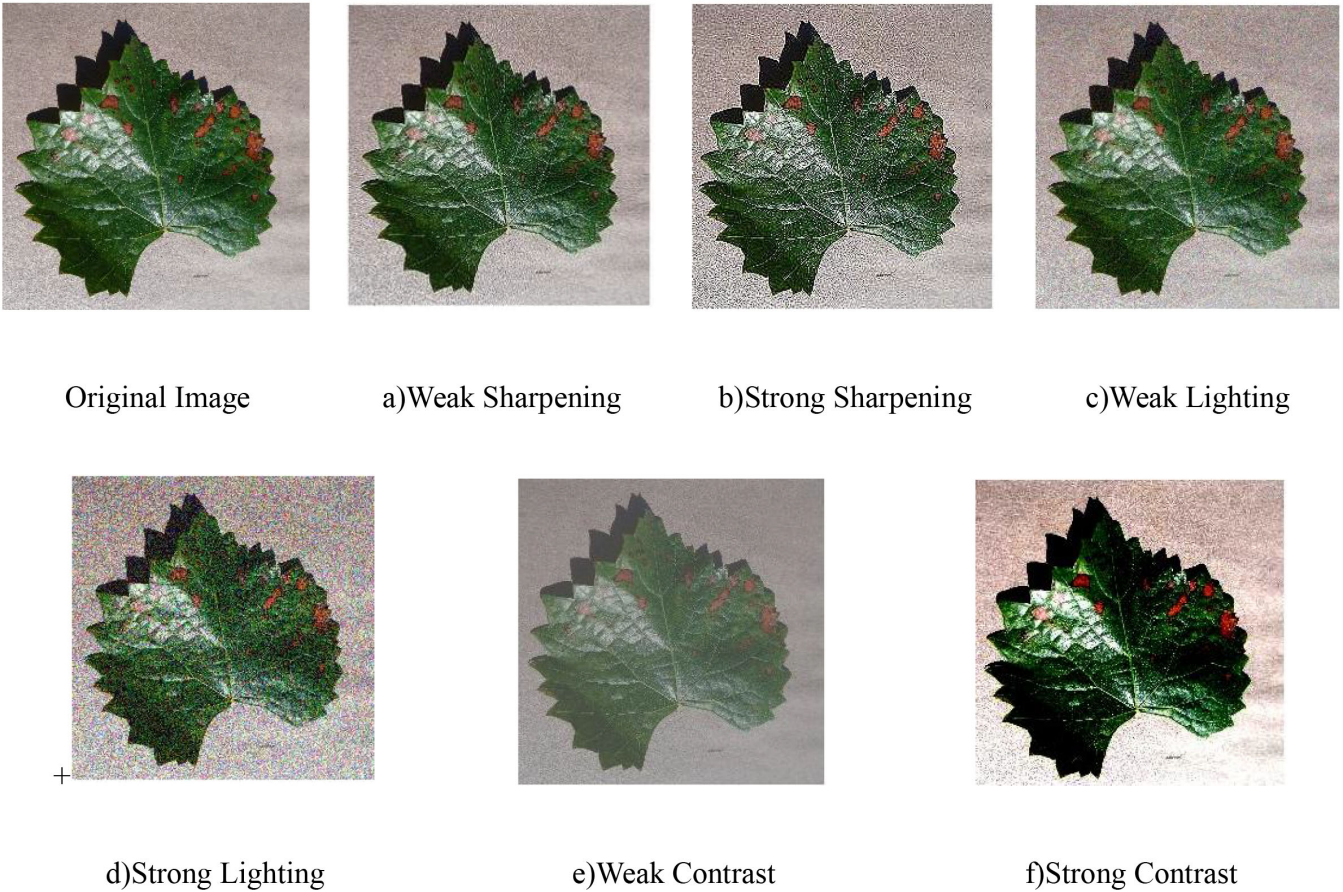
**(a–f)** Schematic diagram after adding different light interference.


The captured images are susceptible to weather conditions, where varying illumination significantly impacts image quality, resulting in diverse performance characteristics regarding sharpness, brightness, and white balance. To better reflect real-world imaging scenarios, this study implements comprehensive data augmentation through parameter adjustments (rotation, cropping, sharpening) on original images’ sharpness, brightness, and contrast, thereby effectively enriching the dataset as demonstrated in **Figures 3a–f**. The image is sharpened using the Laplacian operator to enhance the clarity of image details. By randomly increasing or decreasing the RGB pixel values, the brightness and contrast of the image are adjusted, and the pixel values of the RGB image are denoted as c(x,y) = [R(x,y), G(x,y), B(x,y)], with sharpening performed as shown in Equation 2.

(2) $$\nabla^{2}[c(x,y)]=\begin{array}{l} \nabla^{2}\text{R}(\text{x}.\text{y})\\ \nabla^{2}\text{G}(\text{x},\text{y})\\ \nabla^{2}\text{B}(\text{x},\text{y}) \end{array}$$

### 2.2 Disease identification deep-broad learning network structure design

Deep learning models have high accuracy in image recognition and classification, but they involve a large number of parameters and long training times. In contrast, broad learning networks, with their flat network structure, can accelerate data processing and learning, achieving efficient training speed and strong nonlinear learning capabilities by expanding the width of the network (Wei et al., 2023). However, the number of nodes in the feature and enhancement layers of broad learning networks cannot be determined, leading to poor recognition accuracy and stability.

**Figure 4:**
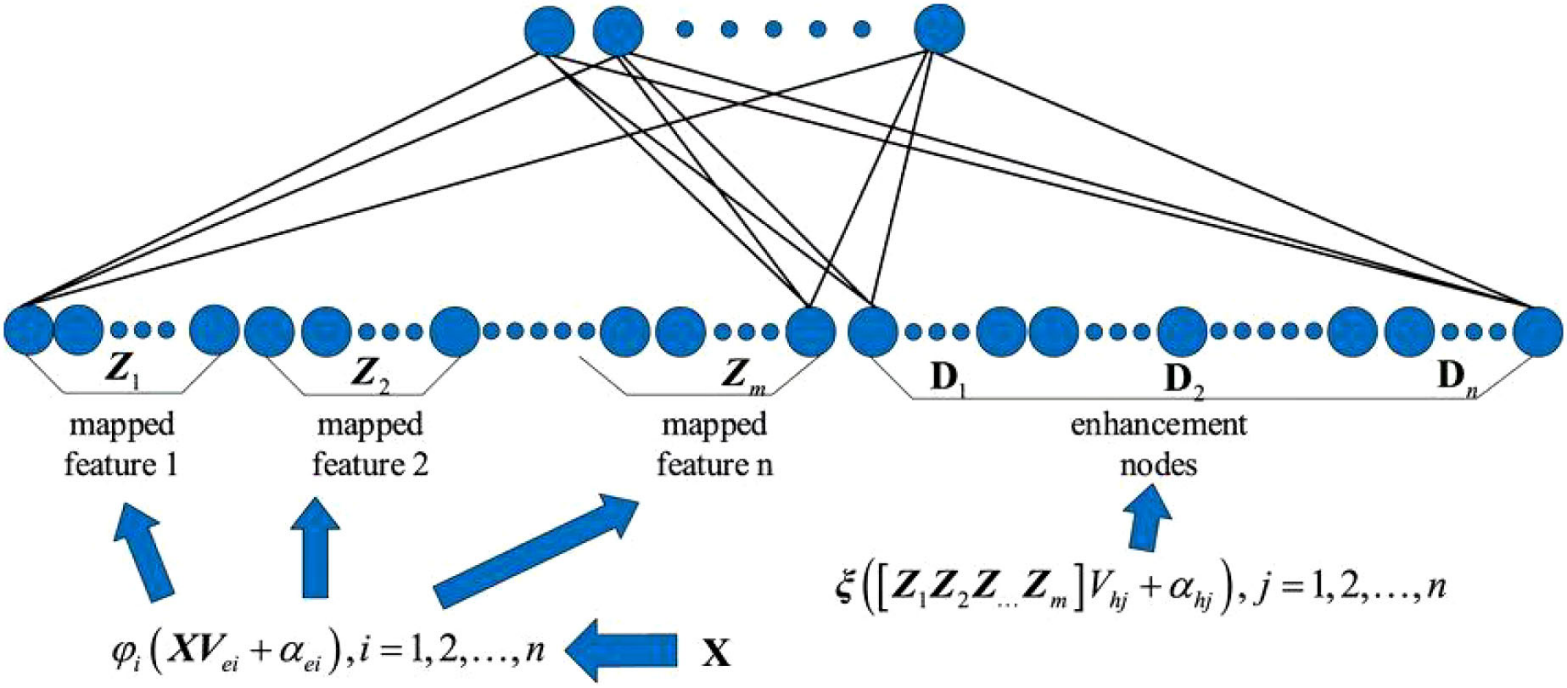
BLS network structure diagram.

Based on the strengths and weaknesses of deep learning and broad learning models, this paper constructs an efficient, adaptive deep-broad learning network structure for grape disease recognition, with broad learning as the core and deep learning mechanisms integrated. This paper employs norm regularization techniques to optimize the loss function of the learning system, replacing the traditional ridge regression method with gradient descent to find the minimum value of the loss function and thereby obtain the optimal output weight matrix. By adopting the compression operation in the SENet attention mechanism, the spatial dimensions of the feature map are compressed to form a feature description that concentrates global information. The weight distribution is performed by analyzing the correlation between feature channels, highlighting important features and suppressing less important ones. This network structure, while ensuring the high learning efficiency of BLS, introduces an adaptive network structure adjustment mechanism that significantly improves the model’s ability to process complex data, enhancing generalization ability, recognition accuracy, and stability, as shown in **Figure 4**.

This paper uses linear mapping functions and input weight matrices to transform the data in order to obtain the mapped feature set. The mapped features are obtained through linear mapping and the activation function as shown in Equation 3:


(3) $$Z_{i}=\emptyset_{i}=(XW_{ei}+\beta_{ei}),i=1,2,3,\ldots ,n$$

$X\in R^{a\times b}$ -input sample data for model training; *a*-total number of samples; *b*-feature dimension of each sample; *W_ei_*-weight matrix for the i feature node; $\emptyset_{i}$ -activation function of the feature node; *β_ei_*-weight matrix connecting the input layer and the mapped feature layer.

After *n* transformations, the final set of mapped features is obtained as shown in Equation 4:


(4) $$Z^{n}=[Z_{1}Z_{2}Z_{3}\ldots Z_{n}]$$

Through nonlinear mapping and activation functions, the mapped nodes are transformed into enhancement nodes as shown in Equation 5:


(5) $$H_{j}=\xi_{j}(Z^{n}W_{hj}+\beta_{hj}),j=1,2,\ldots ,m$$

*H_j_*-the j group of enhancement nodes; *ξ_j_*-activation function; *W_hj_*-the j group of random connection weight matrices; *β_hj_*-the j group of bias matrices. The enhancement nodes obtained through *m* transformations are shown in Equation 6:


(6) $$H^{m}=[H_{1},H_{2},H_{3},\ldots ,H_{m}]$$

The outputs of the feature nodes and enhancement nodes are combined into *A* = [*Z_n_* | *H_m_*]. A is then weighted and mapped to form the network output Y, as shown in Equation 7:


(7) $$\hat{Y}=AW$$

The obtained feature nodes and enhancement nodes are used as inputs to the Least Squares Support Vector Machine (LSSVM), and the corresponding feature vectors are solved. The weight matrix representing the system’s input to output is determined using ridge regression, as shown in Equation 8:


(8) $$W={\left(\lambda I+A^{T}A\right)}^{-1}YA^{T}$$

*λ*-regularization factor; *I*-identity matrix.

This study optimizes the ridge regression structure of BLS using the Adam algorithm, enabling adaptive learning rate adjustment, reducing the need for hyperparameter tuning, and lowering the cost of model selection and evaluation, while improving stability and minimizing the loss function. However, the Adam algorithm suffers from a non-convergence issue, where more weight is given to recent gradients, causing the influence of distant gradients to gradually decay, which hinders effective learning rate adjustment.

**Figure 5:**
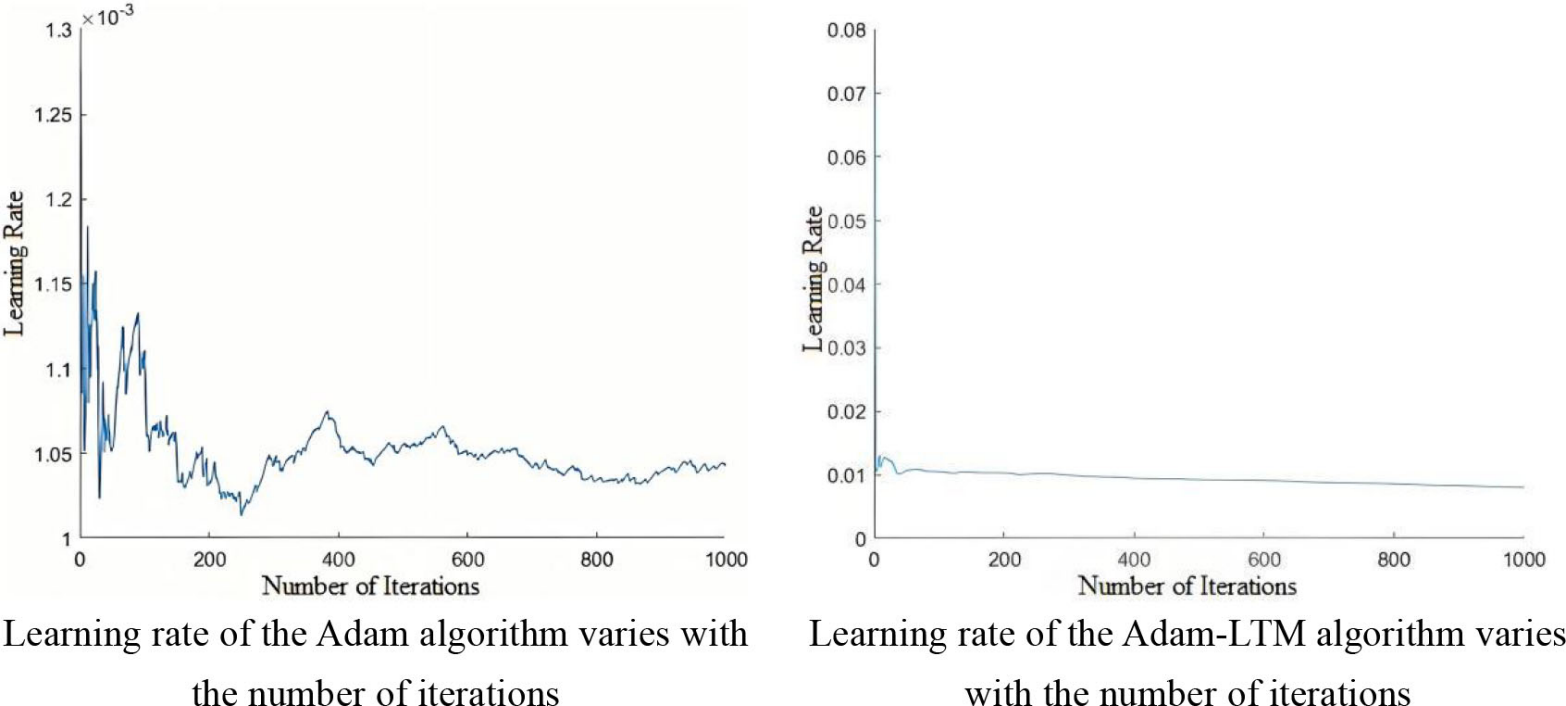
Variation of the learning rate with the number of iterations.


In response to the specific scenario of grape leaf disease, we introduce a decay factor γ to develop the Adam-LTM (Adam with Long-Term Memory) optimization algorithm. This approach weights historical gradient information, enhancing the long-term memory effect. Adam-LTM adjusts the calculation methods of the first- and second-order moments, and controls the decay rate of the moving average index by tuning the decay factor *β*_1_, *β*_2_. The algorithm updates parameters via vector operations, as shown in Equation 9, with a comparison of learning rate stability illustrated in **Figure 5**.

(9) $$\begin{array}{c} g_{t}=f'(x_{t-1})\\ m_{t}=\gamma \cdot m_{t-1}+(1-\gamma )\cdot (\beta_{1}\cdot m_{t-1}+(1-\beta_{1})\cdot g_{t})\\ v_{t}=\gamma \cdot v_{t-1}+(1-\gamma )\cdot (\beta_{2}\cdot v_{t-1}+(1-\beta_{2})\cdot g_{t}^{2}) \end{array}$$

*m_t_*-matrix vector; *g_t_*-gradient at the current time step; $g_{t}^{2}$ -squared gradient; *β*_1_-first-order momentum decay factor; *β*_2_ second-order momentum decay factor; *v_t_*-exponentially weighted infinity norm *γ*-newly introduced decay factor, close to but less than 1.

Since the initial values of *m_t_* and *v_t_* are set to 0, the gradient mean *m_t_*/*v_t_* tends to 0 during the early stages of training. Therefore, in this study, the first-order and second-order moments are bias-corrected starting from the initial time step the function as shown in Equation 10:


(10) $$\begin{array}{c} m_{t}^{'}=\frac{m_{t}}{1-\beta_{1}^{t}}\\ v_{t}^{'}=\frac{v_{t}}{1-\beta_{2}^{t}} \end{array}$$

Where the iterations of the $\beta_{1}^{t}$ , $\beta_{2}^{t}$ algorithm decay over time, i.e., the $\beta_{1}^{t}={\left(\beta_{1}\right)}^{t},\;\beta_{2}^{t}={\left(\beta_{2}\right)}^{t}$ condition.

The parameter iteration update calculation is given by Equation 11:


(11) $$x_{t}=x_{t-1}-\alpha \frac{m_{t}^{'}}{\sqrt{v_{t}^{'}}+\epsilon }$$

*α*-the learning rate hyperparameter, set to 0.001. *ϵ*-the smoothing exponent, set to 10^–8^.

**Figure 6:**
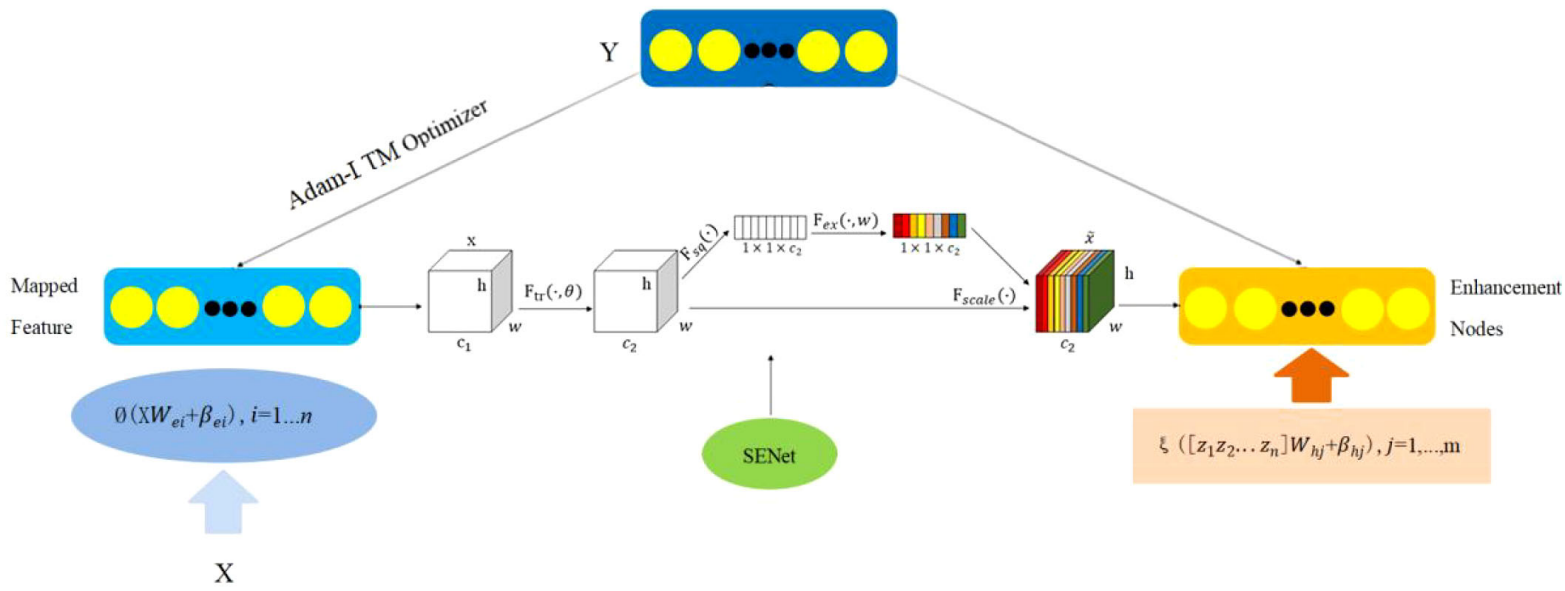
Adam-LTM-BLS structure with embedded SENet module.

Due to the complex structure of grape leaves, this paper integrates the SENet (Squeeze and Excitation Networks) attention mechanism from deep learning into the Adam-LTM-BLS framework, resulting in the ABLSS (Adam-LTM-BLS-SENet) Deep-Broad Learning Network model structure to enhance the ability to extract key features. This study combines the attention mechanism with feature selection in deep learning to enable the model to automatically identify and focus on the most important features, thereby improving performance and reducing interference from redundant features. As a result, the algorithm’s ability to recognize unknown grape leaf disease types or adapt to changing environments is enhanced. The structure is shown in **Figure 6**.

This paper evaluates the importance of each feature through the attention mechanism and dynamically weights or ignores certain features based on this evaluation. The goal of feature selection is to retain the features that have the greatest impact on the final prediction result while ignoring or reducing redundant or unimportant features. The core step of applying the attention mechanism to broad learning is to compute dynamic attention weights for the input features. Feature selection generally aims to reduce the dimensionality of the input data, eliminating features that do not contribute significantly to the model’s prediction. The attention mechanism, however, can assign different weights to each feature based on its dynamic importance in the input data. That is, for each sample’s input, different weights are calculated based on the relative importance of its features, allowing the model to flexibly focus on the most important features for prediction.

Therefore, the input features need to be normalized first. Then, a fully connected layer of a network is used to generate attention weights for each feature, calculating dynamic attention weights for the input features. By feeding the features through a neural network, the attention score for each input feature is computed. Since different features have varying levels of importance, the attention scores are converted into weights. Finally, after normalizing with the Softmax function, important or weighted features are selected.

The ABLSS model applies a squeezing operation to the feature dimensions, transforming the 2D feature map into a real-valued representation. It then uses an activation mechanism to obtain the weight of each feature channel, followed by weighted processing of the feature channels to highlight the importance of each channel, as shown in Equation 12:


(12) $$u_{c}^{\prime }=u_{c}\sigma \{W_{2}\sigma [W_{1}\frac{1}{H\times W}\sum_{i=1}^{H}\sum_{j=1}^{W}u_{c}(i,j)]\}$$

*u_c_*-the c-th channel in the feature map; *W*- width of the feature map; *H*- height of the feature map; *i*, *j*- the pixel value at the i-th row, j-th column in the channel; *W*_1_-the compressed number of channels; *W*_2_-the recovered number of channels; *σ*-ReLU activation function; $u_{c}^{'}$ -the weighted c-th channel after processin.

### 2.3 Improvement of multi-scale feature extraction and segmentation algorithm

**Figure 7:**
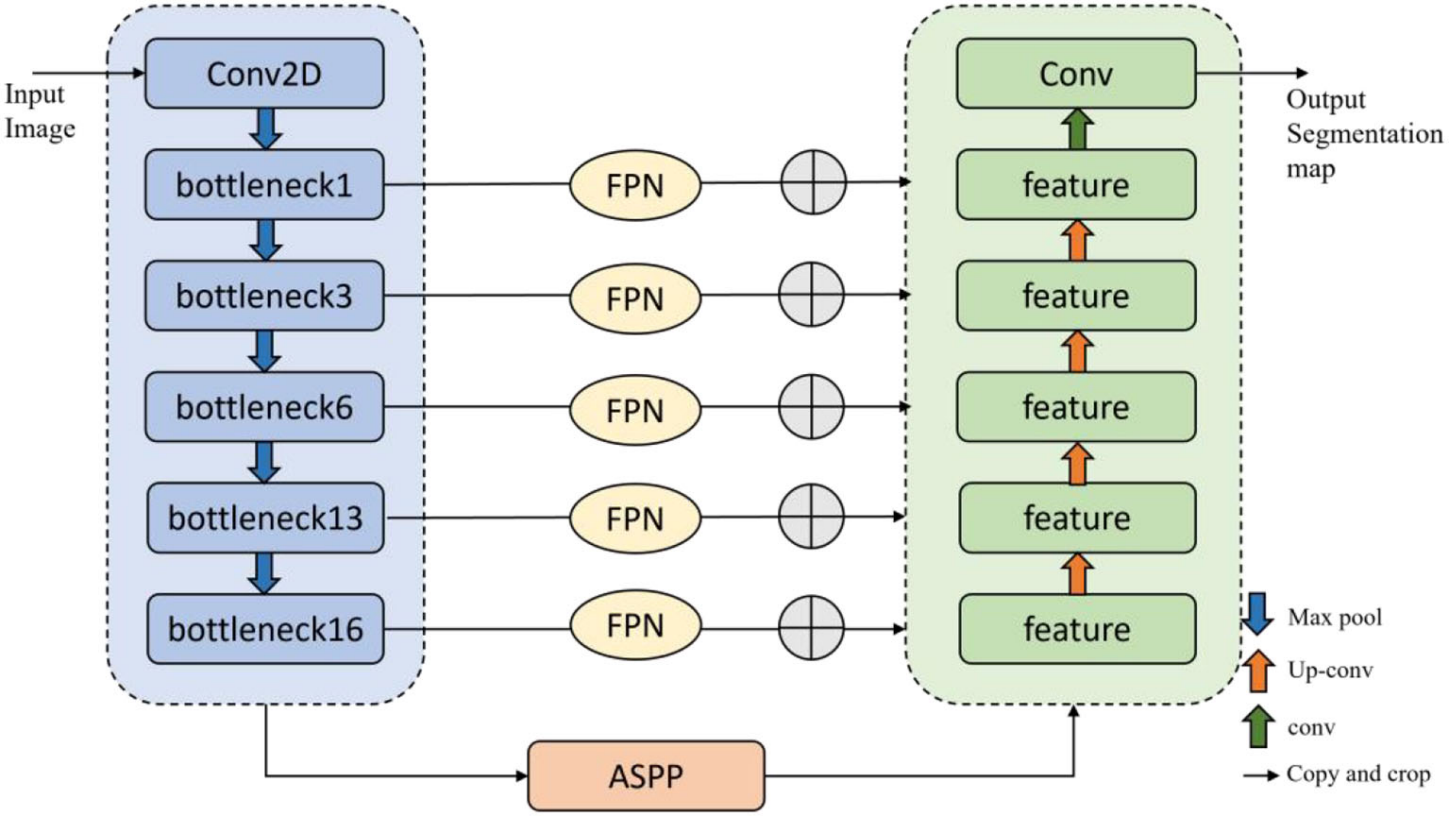
MSCU-Net structure diagram.

The contraction and expansion path structure of the U-Net network can maintain high segmentation accuracy even with limited training samples, effectively handling small-sample segmentation tasks. However, U-Net is prone to losing details, leading to overfitting and increased computational costs. Therefore, this paper proposes a segmentation model based on multi-scale context, MSCU-Net (Multi-Scale Contextual U-Net), to achieve accurate and efficient segmentation of grape beaf blights, as shown in **Figure 7**. This paper integrates Atrous Spatial Pyramid Pooling (ASPP) and Feature Pyramid Networks (FPN) into the original U-Net model to enhance multi-scale information capture and improve the model’s ability to express details.

By adding a series of dilated convolutions with different dilation rates to each branch of the Spatial Pyramid Pooling (SPP), Atrous Spatial Pyramid Pooling (ASPP) performs feature extraction for each dilation rate individually, and then combines the features with the dilated features, as shown in Equation 13:


(13) $$y=Concat(image(X),H_{1,1}(X),H_{6,3}(X),H_{12,3}(X),H_{18,3}(X))$$

*H_r_*,*_n_* (*X*)-Apply dilated convolution on *X* with a sampling rate of *r* and a kernel size of *n*×*n*; *image*(*X*)-Use global average pooling to extract image-level features from the input *X*.

ASPP consists of several parallel dilated convolution branches, each with a different dilation rate, enabling the simultaneous capture of contextual information at multiple scales (Biao et al., 2023). The output *Y_r_* of the dilated convolution layers is given by Equation 14:


(14) $$Y_{r}=\text{ReLU}(\text{BatchNorm}(\text{Conv}(X;k,r)))$$

*X*-the input feature map; *k*-the convolution kernel; *r*-the dilation rate; BatchNorm-accelerates the training process and improves the model’s generalization ability.

The outputs of all dilation convolution branches are fused in the depth direction, as shown in Equation 15:


(15) $$Y_{\text{ASPP}}=\text{Concat}(Y_{1},Y_{2},\ldots ,Y_{N})$$

The output *Y*_ASPP_ is used as input for upsampling. After further adjusting the number of channels through 1×1 convolution, it is fed into the encoder, which allows for better reconstruction of high-quality segmentation maps during the decoding process.

**Figure 8:**
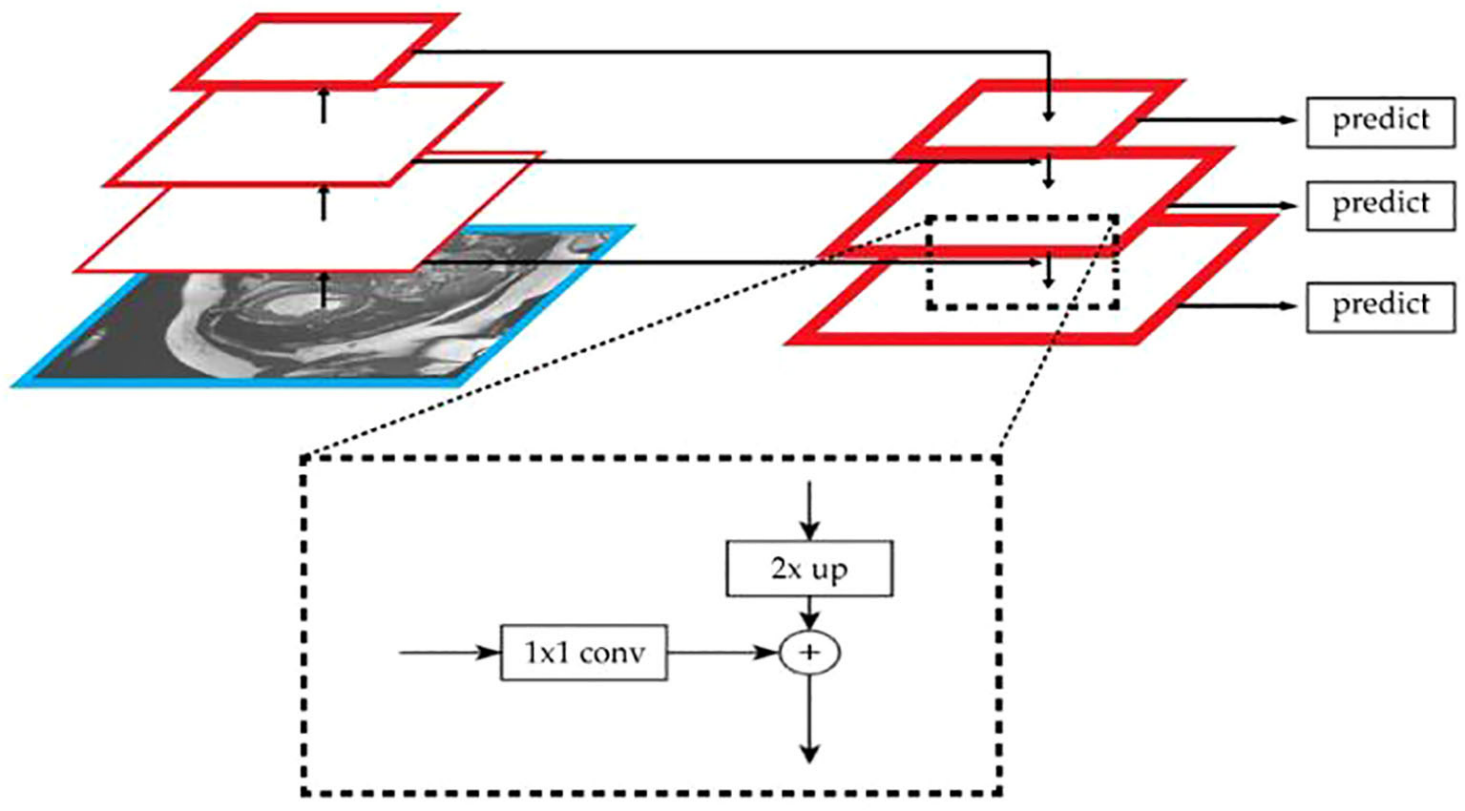
The network architecture of FPN.

To effectively utilize the spatial location information of low-level features and the rich semantic information of high-level features, an additional path from the top layer to the bottom layer is introduced through the FPN (Feature Pyramid Network) algorithm. This significantly enhances the ability to detect objects of different sizes. The network architecture is shown in **Figure 8**.

The algorithm’s bottom-up path extracts and refines image features layer by layer. As the number of layers increases, the size of the feature maps gradually decreases. In this study, the network is divided into different stages based on the size of the feature maps. The stride is set to 2 to ensure that the size of the feature maps at each stage is half of the previous layer. By combining FPN with U-Net, the effect of multi-scale feature fusion is enhanced, thus improving the model’s ability to recognize targets of different sizes in semantic segmentation tasks. The study uses skip connections to directly incorporate the feature maps from each layer of the encoder into the decoder stage. For the output of each encoder layer, 1×1 convolution is applied to reduce the number of channels in the feature map. Finally, the upsampled feature maps are fused with the feature maps of the decoder layer, as shown in Equation 16:


(16) $$D_{i}^{'}=\text{Merge}(E_{i}^{'},D_{i})$$

$D_{i}^{'}$ -the generated fused feature map; $E_{i}^{'}$ -the feature map from the encoder; *D_i_*-the feature map from the decoder.

The fused feature map is then further upsampled and convoluted until it is restored to the same resolution as the input image. Finally, a 1×1 convolution is applied at the output layer to convert the feature map into the predicted segmentation map.

### 2.4 Grape leaf disease diagnosis method

The classification of grape diseases provides a basis for precise pesticide and fertilizer management. In this study, disease grading is performed by calculating the ratio of diseased pixel points to the total leaf pixel points, as shown in Equation 17:


(17) $$p_{a}=\frac{N_{i}}{N_{I}}\times 100\%$$

*p_a_*-the percentage of the leaf area affected by the disease; *N_i_*-the number of diseased pixel points in the image; *N_I_*-the total number of pixel points in the entire leaf area.

Due to the lack of a clear grading system for grape diseases in the industry, this study uses the Analytic Hierarchy Process (AHP) to determine the disease severity levels. The judgment matrix of the criterion layer for the objective layer is shown in **Table 2**, and the scoring evaluation for the alternative layer is shown in **Table 3**.

**Table 2:** Judgment matrix.

	Disease Type	Lesion area percentage
Disease Type	*e*_11_	*e*_12_
Lesion area percentage	*e*_21_	*e*_22_

**Table 3:** Score evaluation table.

Alternative Layer	C0	C1	C2	C3	C4
Scoring Criteria	1	3	5	7	9

The maximum eigenvalue *λ_max_* of each judgment matrix and its corresponding normalized eigenvector are calculated as shown in Equation 18:


(18) Ax=λx

*A*-the judgment matrix; *λ*-the eigenvalue; *x*-the corresponding eigenvector. The eigenvector corresponding to the maximum eigenvalue represents the weight.

The consistency index (*CI*) is calculated as shown in Equation 19:


(19) $$CI=\frac{\lambda_{max}-n}{n-1}$$

*n*-the order of the judgment matrix.

The consistency ratio (*CR*) is calculated as shown in Equation 20:


(20) $$CR=\frac{CI}{RI}$$

*RI*- the random consistency index, which depends on the order of the judgment matrix. If *CR* < 0.1, the judgment matrix is considered consistent; otherwise, the matrix needs to be re-evaluated.

To calculate the weights of the alternative layer under different criteria, the weights of the alternative layer under each criterion are combined and ranked. The weight of the alternative layer under each criterion is multiplied by the weight of that criterion, as shown in Equation 21:


(21) $$W_{C_{i}}=(W_{C_{i}|B1}\times W_{B1})+(W_{C_{i}|B2}\times W_{B2})$$

*W_B_*_1_, *W_B_*_2_-the weights of the goal laye, and the weights of the alternatives in the alternative layer under the two criteria layers are denoted as $W_{C_{i}|B1}$ and $W_{C_{i}|B2}$ ; $W_{C_{i}}$ -the *C_i_* overall weight of the alternative.

The global consistency ratio (*GCR*) is calculated as shown in Equation 22:


(22) $$GCR=\frac{\left(CI_{B1}\times W_{B1})+(CI_{B2}\times W_{B2}\right)}{RI}$$

If *GCR* < 0.1, the consistency of the model is considered acceptable.

This study surveyed 200 grape farmers and 20 experts to obtain the judgment matrices and established the grape grading standards, as shown in **Tables 4** and **5**.

**Table 4:** Judge the specific value of the matrix.

	Disease type	Lesion area percentage
Disease type	1	0.33
Lesion area percentage	3	1

**Table 5:** Grape leaf disease classification standard.

Disease grade	Grape Black Rot lesion area percentage	Grape Black Measles lesion area percentage	Grape Leaf Blight lesion area percentage
Level 1	≤10%	≤5%	≤15%
Level 2	(10%-25%]	(5%-20%]	(15%-0%]
Level 3	(25%-50%]	(25%-45%]	(30%-55%]
Level 4	≥50%	≥45%	≥55%

## 3 Experiments and results analysis

### 3.1 Experimental design

The experimental environment in this paper is a Windows 10 operating system, equipped with an Intel(R) Core(TM) i7-8750H CPU (2.20GHz). The GPU configuration includes Intel(R) UHD Graphics 630 (8GB of VRAM) and an RTX 1070 (8GB of VRAM). The system has 16GB of memory, and the programming language used for the experiment is Python 3.10.

#### 3.1.1 Experimental purpose and methods

To test the effectiveness of the proposed algorithm and the significance of the optimization scheme on performance improvement, experiments were conducted on the ABLSS deep learning model and the improved MSCU-Net algorithm.

##### 3.1.1.1 Performance experiment of the ABLSS deep learning network model

This experiment includes stability experiments, convergence experiments, and recognition accuracy experiments. In the experiment, the regularization parameter of the ABLSS model is set to *λ* = 10^–10^, and the mapping parameters *W_ei_* and *β_ei_* are randomly selected from the standard uniform distribution in the range [-1, 1]. *β*_1_ = 0.9, *β*_2_ = 0.99, stability constants *ϵ* = 10^–8^, with the initial learning rate set to 0.001, and the number of iterations set to *T* = 100. The number of gradient descent iterations is set to 100 epochs, with the number of feature nodes set to 40, and the number of enhancement nodes set to 500.

This paper analyzes the change in model accuracy by altering the number of feature nodes and enhancement nodes in the model. By comparing the changes in accuracy, the stability of the model is evaluated. The Black_rot dataset is used for the experiment, and the number of enhancement nodes is incrementally increased based on different feature node counts. Specifically, the number of feature nodes is 10, 40, 70, and 100; the number of enhancement nodes ranges from 100 to 1000, with an increment of 100 nodes each time. The convergence experiment evaluates the convergence of the algorithm by analyzing the number of iterations required to converge to the minimum loss function value during the iterative process. In the recognition accuracy experiment, 10-dimensional feature values are used as the input to the classifier, and recognition accuracy is analyzed. Each experiment is repeated 10 times, and the average of the 10 results is taken as the effective value.

##### 3.1.1.2 MSCU-Net algorithm performance experiment

This experiment includes comparison experiments on the loss functions of the improved model, comparison of average segmentation accuracy, and comparison of segmentation accuracy for different disease types. The performance of the proposed model is compared with the original U-Net, FCN, and SegNet models. The initial learning rate is set to 0.001, the batch size for each training session is set to 2, and the number of iterations is 100. The convergence of the models is verified by analyzing the loss function curves of the U-Net and MSCU-Net models.

#### 3.1.2 Model evaluation metrics

This study evaluates the speed and performance of the model using two metrics: mean intersection over union (MIOU) and mean pixel accuracy (MPA). MIOU effectively reflects the accuracy of lesion segmentation, as shown in Equation 23.


(23) $$M=\frac{1}{k+1}\sum_{i=0}^{k}\frac{p_{ii}}{\sum_{j=0}^{k}p_{ij}\;+\;\sum_{j=0}^{k}p_{ji}-p_{ii}}$$

*M*-the MIOU result; *k*-the number of foreground objects; *p_ii_*-the number of pixels correctly predicted as class *i*-th;

*p_ji_*-the number of pixels that actually belong to class *i* but are predicted as another class *j*.

MPA is the average pixel accuracy of each classification category, as shown in Equation 24.


(24) $$A=\frac{1}{k+1}\sum_{i=0}^{k}\frac{p_{ii}}{\sum_{j=0}^{k}p_{ij}}$$

*A* -the calculation result of the mean pixel accuracy (MPA).

### 3.2 ABLSS model performance experiment results and analysis

#### 3.2.1 Stability experiment

**Figure 9:**
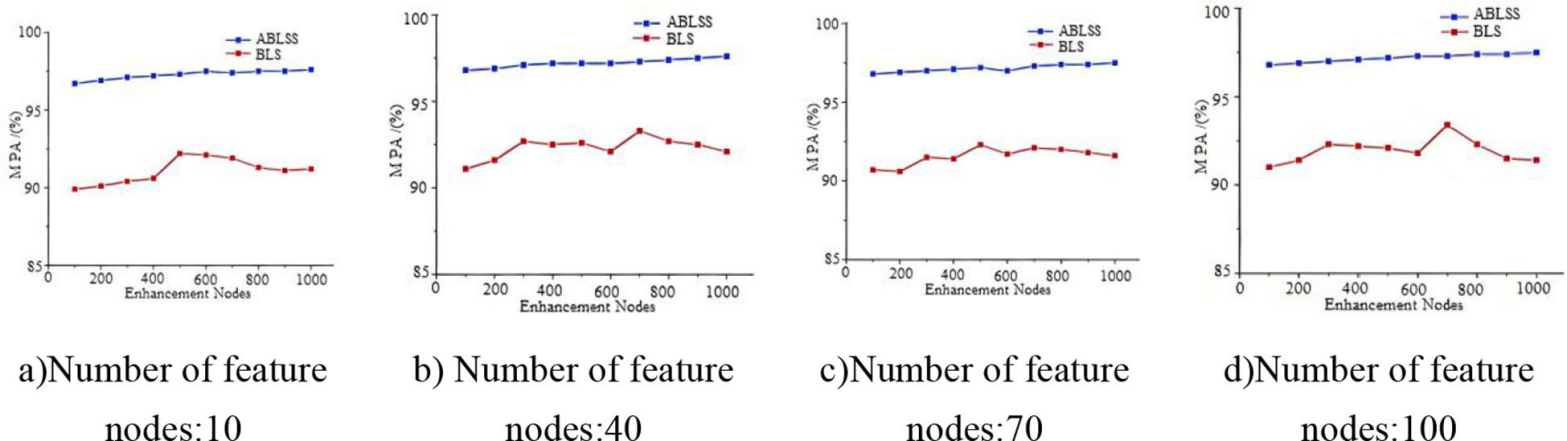
**(a–d)** Test accuracy under four different number of node counts: 10, 40, 70, 100.

As shown in **Figures 9a–d**, the accuracy of ABLSS is significantly higher than that of BLS. Moreover, as the number of enhancement nodes increases, the accuracy of ABLSS steadily improves, while the accuracy of BLS fluctuates significantly without showing a clear upward trend. This paper demonstrates that the Adam-LTM optimization algorithm-based improvement method for BLS can significantly enhance the algorithm’s ability to analyze and extract fine features from complex images, improving both stability and accuracy. Thus verifying the correctness and effectiveness of optimizing the ridge regression structure of the Broad Learning System (BLS) using the Adam algorithm to enhance stability, as well as the correctness and effectiveness of the proposed improvement—introducing an additional decay factor γ, which enables Adam-LTM to enhance the retention of historical information and improve long-term memory by adjusting the computation of the first- and second-order moments through the decay factor.

#### 3.2.2 Convergence experimental

**Figure 10:**
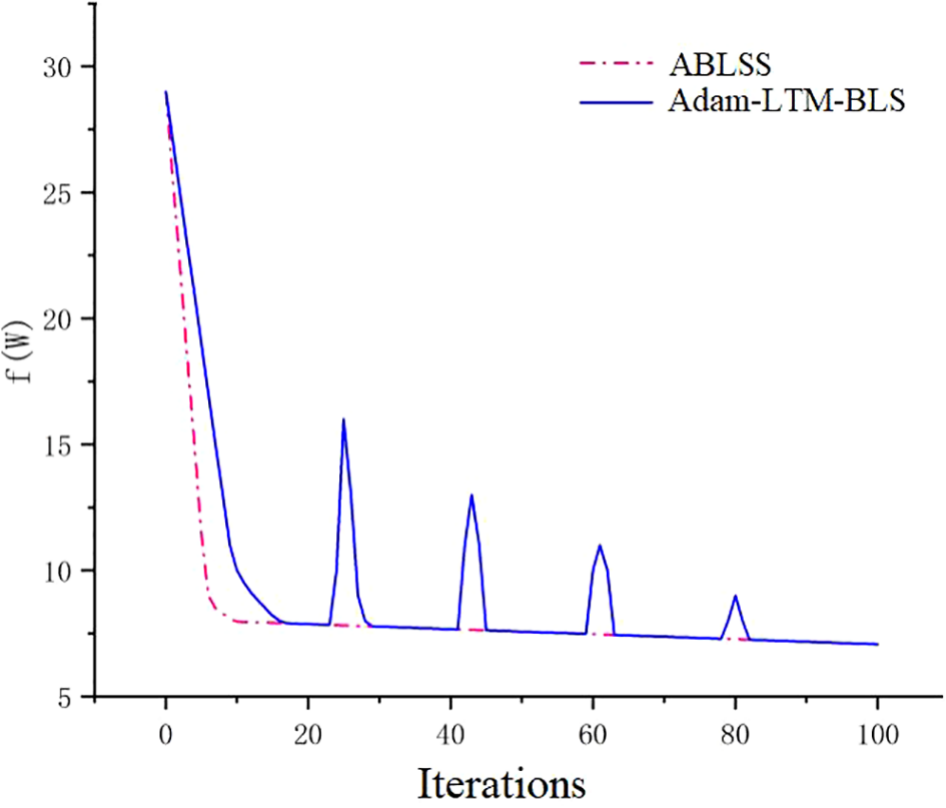
Comparison of loss function convergence for gradient descent-based algorithms.

From **Figure 10**, it can be observed that for the Black_rot dataset, both Adam-LTM-BLS and ABLSS are able to converge to the most optimal result over time. However, Adam-LTM-BLS exhibits oscillation during the convergence process, with the curve showing a sawtooth pattern, stabilizing around the 100th iteration. This indicates poorer stability and worse convergence. On the other hand, the convergence curve of ABLSS is smoother, stabilizing within just 10 iterations, reaching a better result. This demonstrates that ABLSS has better convergence performance, with higher stability and efficiency. Adam-LTM-BLS cannot achieve an optimal value with fewer iterations. This paper demonstrates that by integrating the SENet module of deep learning into Adam-LTM-BLS, the algorithm’s ability to analyze complex images and extract key features is significantly enhanced, enabling it to achieve optimal values with fewer iterations. This validates the correctness and effectiveness of the proposed improvements to the deep learning network. The experimental results validate the effectiveness of our proposed enhanced approach that integrates deep learning mechanisms into the broad learning system (BLS) framework. This hybrid methodology synergistically combines the complementary advantages of BLS and deep learning, achieving significant improvements in both recognition accuracy and efficiency.

#### 3.2.3 Recognition accuracy experiment

**Figure 11:**
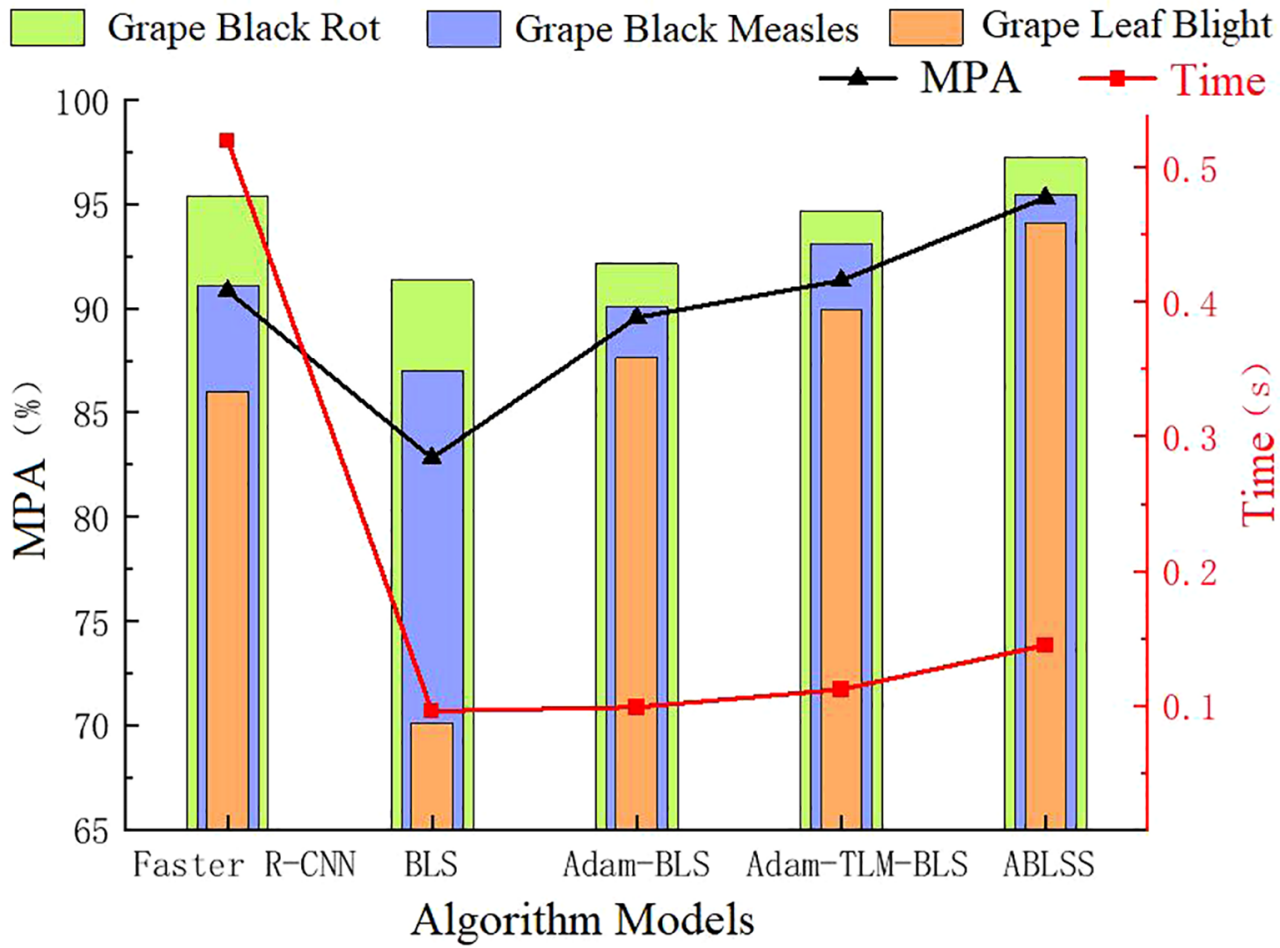
Performance comparison of different algorithm models.

From **Figure 11**, it can be seen that ABLSS achieves the highest recognition accuracy for all three types of grape leaf diseases. The average recognition accuracy is 7.69% higher than that of BLS and 4.48% higher than that of the deep learning network Faster R-CNN, showing a significant improvement. This proves the correctness and effectiveness of the proposed improvements to the deep learning network. From the table, it is evident that BLS has the lowest accuracy, 3.21% lower than Faster R-CNN. However, after the improvement using the Adam-LTM algorithm in this paper, the accuracy increases by 3.71%, surpassing the deep learning network. As shown in the **Figure 11**, the error bars indicate a low degree of data dispersion, with values closely clustered around the mean, demonstrating high data reliability. The recognition accuracy of BLS, Adam-BLS, Adam-LTM-BLS, and ABLSS gradually increases, effectively addressing the issue of poor accuracy and stability in BLS, proving the effectiveness of Adam-LTM algorithm for width learning improvement.

Broad learning significantly outperforms deep learning in terms of recognition speed. Although the improvements in this paper cause a gradual increase in the recognition algorithm’s time, the time curve shows that the curves for BLS, Adam-BLS, Adam-LTM-BLS, and ABLSS are relatively flat. ABLSS is only 0.049s slower than BLS, indicating that the proposed method has minimal impact on the recognition efficiency of BLS. Compared to deep learning networks, ABLSS is 0.375s faster, an improvement of 72.12%, effectively addressing the slow recognition speed problem of deep learning. This proves that the ABLSS deep learning network model proposed in this paper not only significantly improves recognition accuracy but also significantly enhances recognition efficiency, validating the effectiveness and correctness of the fusion method between broad learning and deep learning.

### 3.3 MSCU-Net algorithm performance experiment results and analysis

#### 3.3.1 Comparison experimental of loss function of the improved model

**Figure 12:**
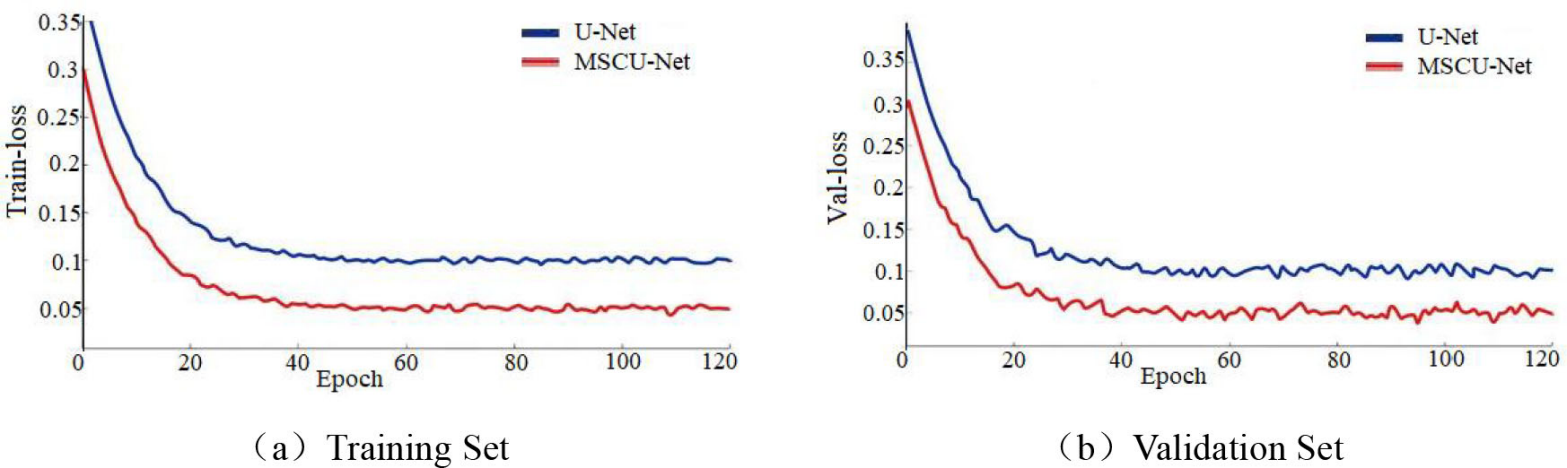
**(a, b)** The comparison of loss functions between training set and validation set before and after U-Net network improvement.

From **Figure 12a**, the loss function change in the training dataset shows that the blue convergence curve stabilizes at approximately 0.098, and the red curve stabilizes at approximately 0.051. This indicates that the improved model fits the training set better than the original model. From **Figure 12b**, the loss function change in the validation dataset shows that the blue convergence curve stabilizes at approximately 0.112, while the red curve stabilizes at approximately 0.049. This indicates that the improved model has significantly better generalization ability. This proves that the proposed method can significantly enhance both the fitting ability and convergence of the model.

#### 3.3.2 Comparison experimental of average segmentation accuracy

**Figure 13:**
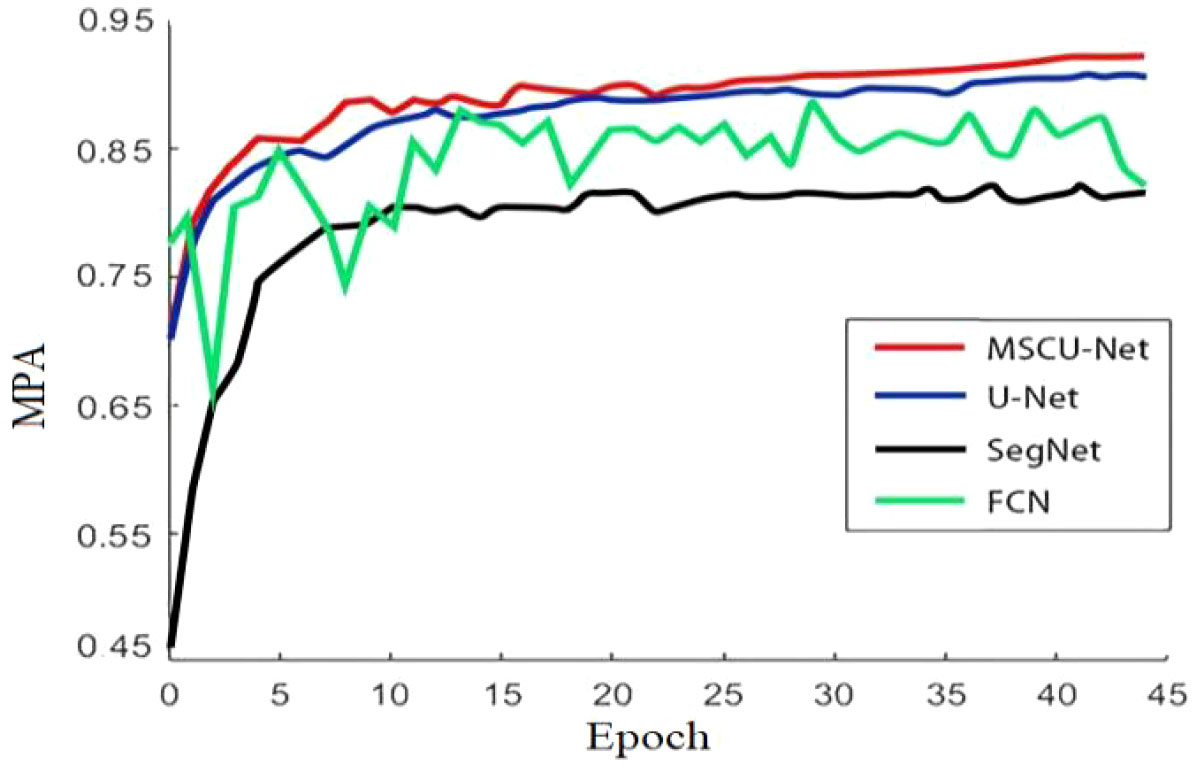
The accuracy comparison line chart of different segmentation models.

From **Figure 13**, it can be seen that with the increase in the number of iterations, the accuracy of all models shows an upward trend. However, the improved MSCU-Net achieves the highest accuracy, significantly outperforming the pre-improved U-Net model, SegNet model, and FCN model. Moreover, the growth curve is the smoothest, indicating that this algorithm has the best stability. This paper demonstrates that by integrating the improved ASPP and FPN models, it can effectively analyze images to construct high-quality segmentation maps, enhancing multi-scale information capture and the model’s ability to express fine details.

**Figure 14:**
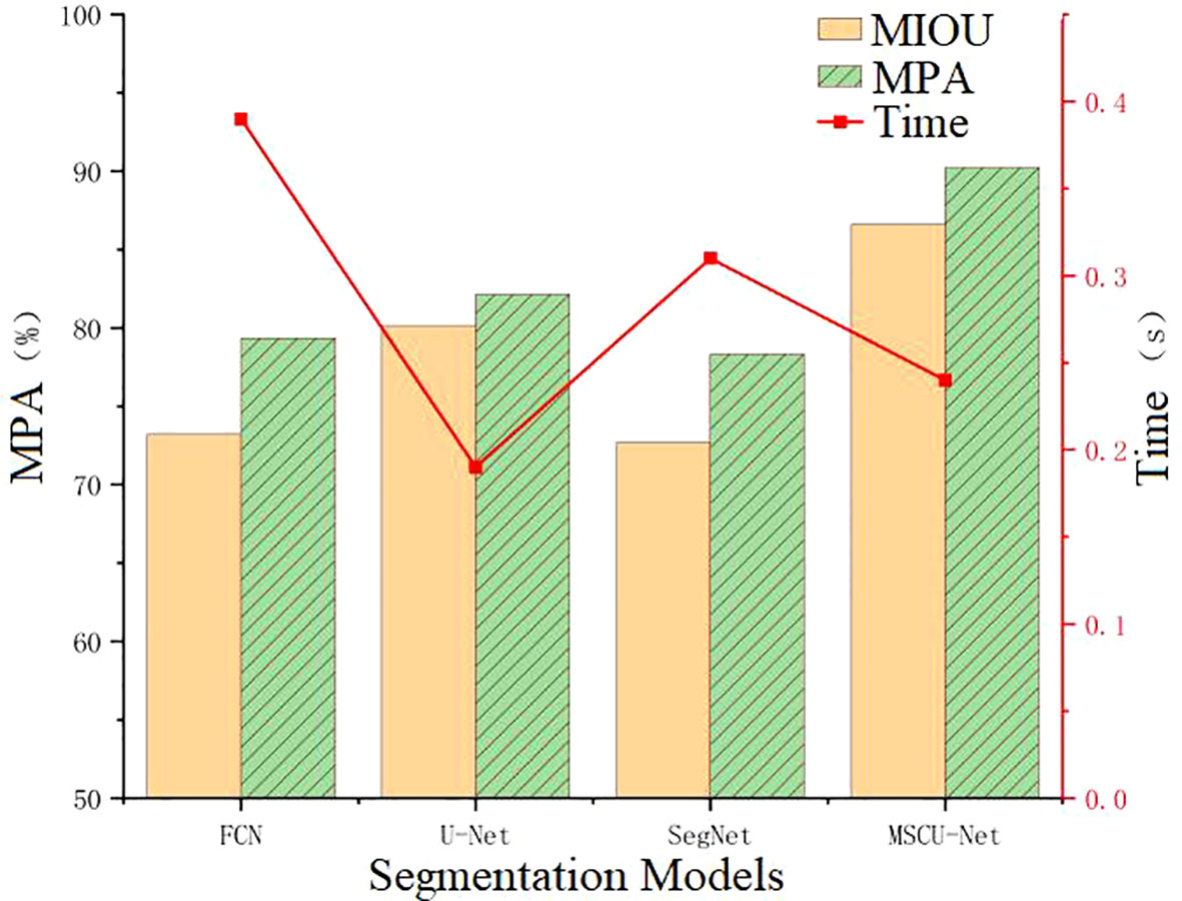
Performance comparison of different models.

According to **Figure 14**, the improved MSCU-Net in this paper achieves the highest MIOU index value, surpassing U-Net, FCN, and SegNet by 6.48%, 13.4%, and 13.92%, respectively. MSCU-Net also achieves the highest MPA index value at 90.23%, which is 8.09%, 10.9%, and 11.91% higher than U-Net, FCN, and SegNet, respectively. This indicates that MSCU-Net is more accurate and reliable in image recognition. Although the image processing time for MSCU-Net is 0.24 seconds, which is not the fastest, it is only 0.05 seconds slower than U-Net, with a relatively small difference. However, its accuracy shows a significant advantage. As shown in the **Figure 14**, the error bars indicate a low degree of data dispersion, with values closely clustered around the mean, demonstrating high data reliability. Therefore, the improved MSCU-Net model proposed in this paper demonstrates superior efficiency and accuracy in the grape leaf disease spot segmentation scenario. The effectiveness of the multi-scale context-based segmentation model MSCU-Net was verified, demonstrating its significant advantages in capturing multi-scale information and enhancing the model’s ability to represent fine details.

**Figure 15:**
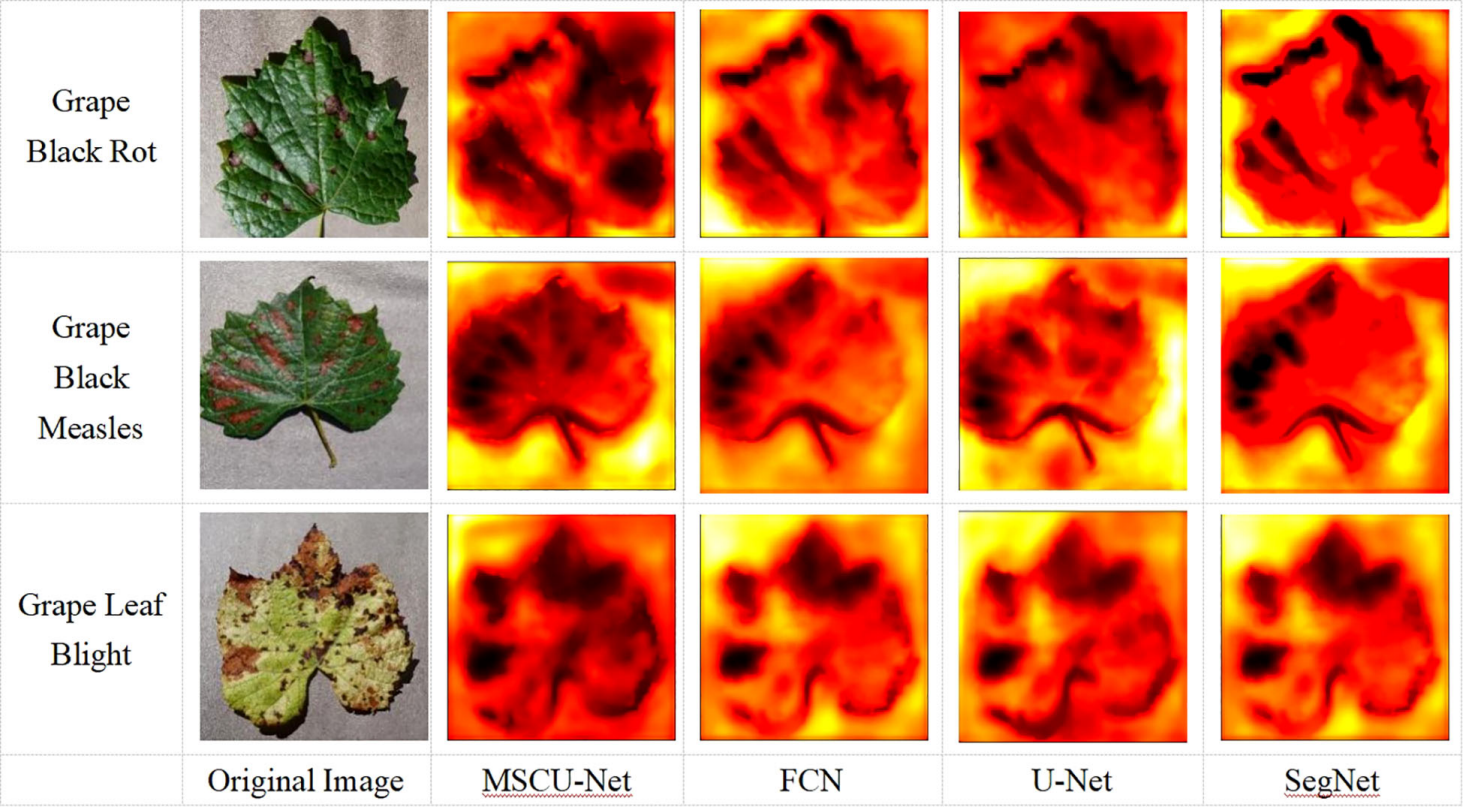
Attention distribution map.

To more intuitively show the model’s activation patterns and attention distribution on disease segmentation in the images, this paper outputs a visual heatmap to observe the model’s attention distribution, as shown in **Figure 15**.

As shown in **Figure 15**, the distribution of disease spots in the original grape leaf image is similar to the black regions in the heatmap, indicating that when recognizing and segmenting the grape leaves, the model’s main attention is focused on the disease spots. The attention distribution of the improved MSCU-Net model is more aligned with the disease spot distribution, demonstrating that the MSCU-Net model can more accurately recognize the disease spot areas and has better coverage of the surrounding regions. This proves that the method of increasing a series of dilated convolutions with different dilation rates in each branch through ASPP, which extracts features at each dilation rate, can enhance the multi-scale contextual information reading capability. This validates that the proposed improvement can better capture the features of the disease spots on the leaves, improving the accuracy and robustness of both recognition and segmentation.

#### 3.3.3 Comparison experimental of segmentation accuracy for different disease types

**Figure 16:**
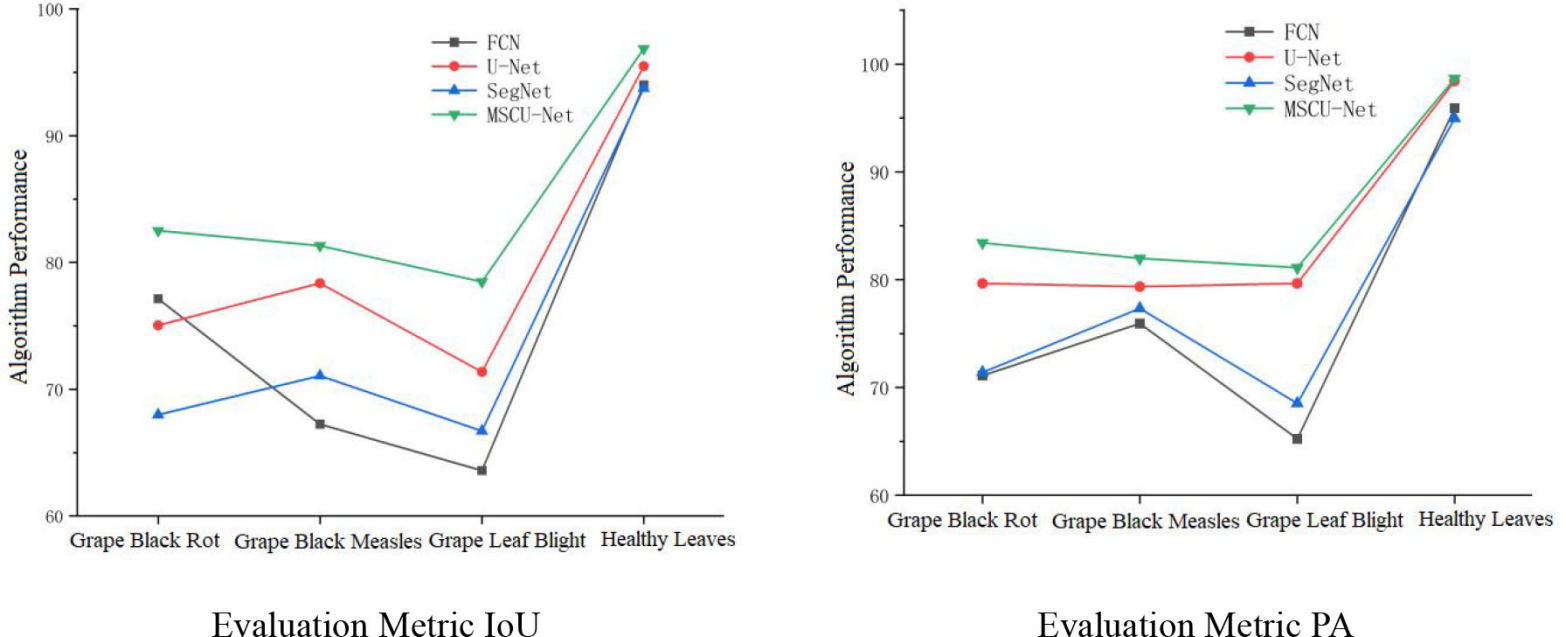
The performance of different models to deal with different lesions.

As shown in **Figure 16**, the four algorithms exhibit similar segmentation results for healthy leaves, but the IoU and PA metrics of the improved MSCU-Net model are significantly higher for the segmentation precision of the three types of disease spots compared to the other three algorithms. This proves that MSCU-Net has a significant advantage in accurately locating and recognizing different types of lesions.

**Figure 17:**
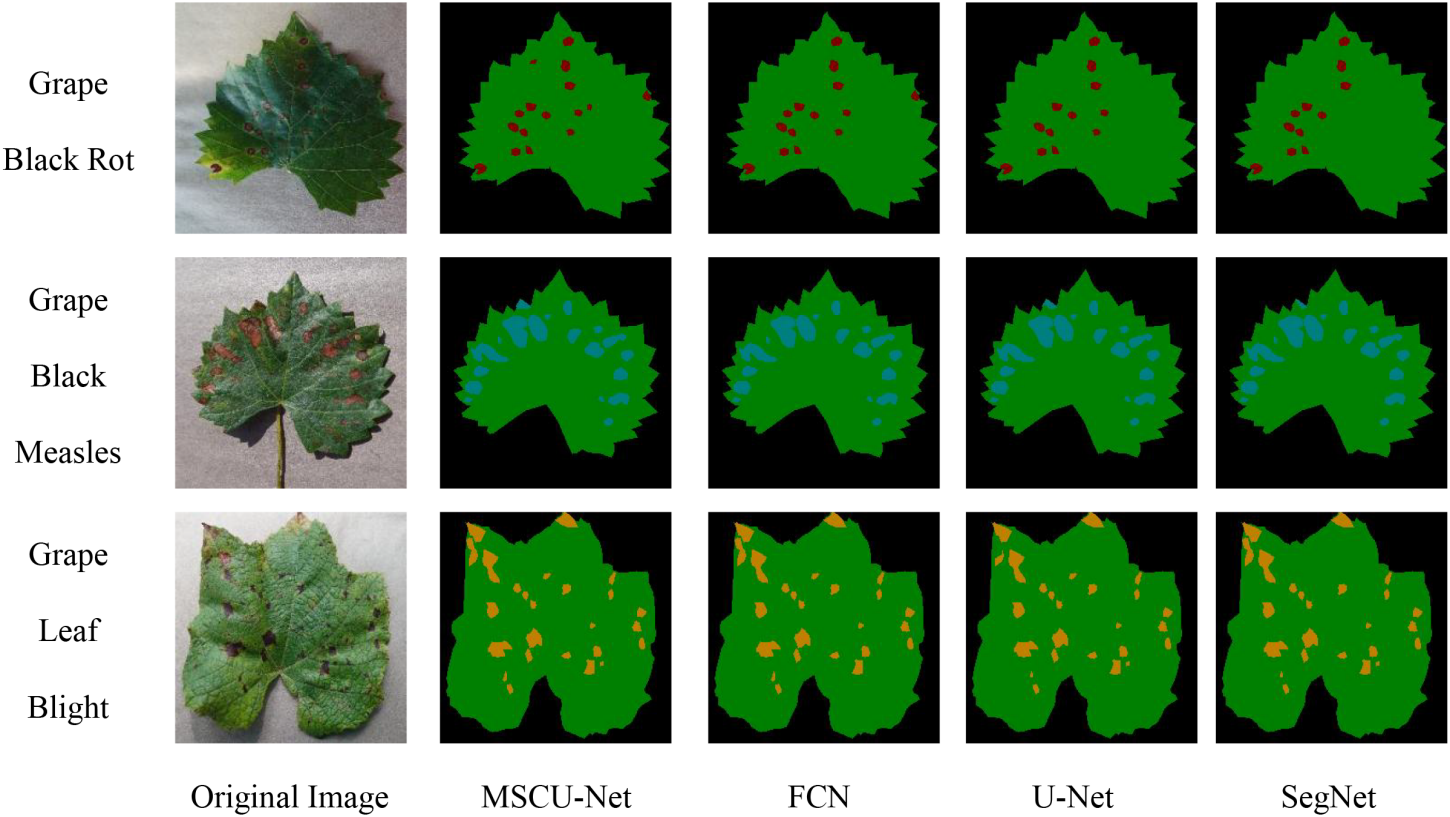
Segmented result of the three diseases by the algorithms.

The IoU and PA metric curves for MSCU-Net’s segmentation precision of the three disease spots are relatively smooth, while the other models show poor stability in the segmentation results for the three disease spots, with both metrics fluctuating. In the IoU metric, SegNet performs better for the black rot lesion segmentation, while U-Net performs better for the grape black measles segmentation, but both models perform poorly for the other two lesions. In the PA metric, SegNet and FCN show large fluctuations, while U-Net’s curve remains more stable. This validates that the improvements made to MSCU-Net are significant. While maintaining the stability of U-Net, it significantly enhances the accuracy and stability of image feature segmentation, with the segmentation results shown in **Figure 17**.

As shown in **Figure 17**, MSCU-Net provides more detailed segmentation, especially with a significantly higher sensitivity to smaller lesions compared to other algorithms, enabling more precise segmentation of small lesions. The segmentation results from FCN and SegNet are relatively poor, while U-Net performs slightly better than FCN and SegNet, but still misses small lesions. This proves that the improved MSCU-Net model performs better in leaf and lesion segmentation, particularly excelling in segmenting small lesions and smoothing the lesion boundaries.

Based on the segmented pixel areas of the leaves and lesions, the disease severity of the leaves is determined by calculating the relative area ratio, as shown in **Table 6**. Based on the disease severity levels, targeted and precise treatments can be carried out to improve grape yield and quality.

**Table 6:** Different leaf disease grades.

leaf parameter	leaf (a)	leaf (b)	leaf (c)
Number of Lesion Pixels	1147	3714	3032
Number of Leaf Pixels	30896	28205	42569
Relative Area Proportion	3.71%	13.17%	7.12%
Disease Grade	Level 1	Level 2	Level 1

### 3.4 Discussion

As shown in **Figure 9**, the accuracy of ABLSS is significantly higher than that of BLS. It is verified that the ridge regression structure of BLS is optimized by Adam algorithm, and the calculation method of first-order and second-order moments is adjusted by adding attenuation factor γ, which significantly improves the ability to maintain historical information. As shown in **Figure 10**, the convergence curves of the two algorithms completely overlap around the 85th iteration. However, the convergence curve of Adam-LTM-BLS exhibits a sawtooth pattern and becomes stable only around the 100th iteration, while the convergence curve of ABLSS is smoother and stabilizes around the 10th iteration, the number of iterations is reduced to 10%. This demonstrates that the optimization of BLS within the broad learning framework can achieve higher recognition accuracy with a sufficient number of iterations. However, although the Adam algorithm improves recognition accuracy, its non-convergence issue causes the recent gradients to be given higher weights, leading to a gradual decay in the impact of distant gradients and ineffective learning rate adjustment. After introducing the Long-Term Memory (LTM) mechanism, while the decay speed of the moving average exponent is effectively controlled, the impact of the Adam algorithm cannot be completely eliminated, resulting in a stepwise improvement in recognition accuracy and a sawtooth-shaped convergence curve.

By integrating the SENet module of deep learning into Adam-LTM-BLS, not only are the negative impacts of the Adam algorithm eliminated, but convergence is further improved. The SENet attention mechanism significantly enhances the ability to analyze complex images and extract key features. When combined with the LTM mechanism, it further optimizes the loss function, achieving optimal weight distribution for all channels in the global feature description, highlighting important features while suppressing less significant ones. The optimal weight allocation is completed during the first stage of Adam-LTM-BLS, which not only significantly improves recognition efficiency but also greatly enhances recognition accuracy.

As shown in **Figure 11**, the five network models achieve the best recognition results for black rot, second best for grape black measles, and worst for grape beaf blight. Analyzing the lesion features, it can be observed that black rot lesions are relatively regular with clear features, making them easier to extract and recognize. Grape black measles are oblong and slightly more complex than the round lesions of black rot, while grape beaf blights are small and irregular, requiring the model to accurately distinguish finer differences, making feature extraction more challenging.

Traditional broad learning significantly lags behind deep learning in recognition accuracy, and as the complexity of the lesions increases, the accuracy gap becomes more evident, with differences of 4.06%, 4.11%, and 15.89%, respectively. This proves that deep learning is more suitable for recognizing complex images. After optimizing broad learning with the Adam algorithm and introducing the LTM mechanism without combining deep learning, the recognition accuracy for black rot is still weaker than deep learning, but the recognition accuracy for grape black measles and grape beaf blight is noticeably better than deep learning. Black rot is the least challenging to recognize, while grape beaf blight is the most difficult, indicating that the optimized broad learning model significantly enhances the ability to extract and analyze features from small and irregular complex images, improving recognition accuracy and solving the limitations of Broad Learning in recognizing complex images. It verifies that the optimization method proposed in this paper can correctly and significantly improve the analysis and recognition accuracy of complex images by Broad Learning.

After Adam-LTM optimization, combined with deep learning network structures and incorporating the attention mechanism, the recognition accuracy for the three diseases is significantly improved, with black rot recognition accuracy even surpassing deep learning models. This demonstrates that the proposed optimization method for broad learning can significantly improve recognition accuracy. Although the improved method leads to a slight increase in recognition time, with ABLSS being only 0.049s slower than BLS, it is still 0.375s faster than deep learning networks. This proves that the proposed ABLSS deep learning model inherits the advantages of high recognition accuracy from deep learning and fast processing speed from broad learning.

## 4 Conclusion

This paper addresses the problem of precise recognition of different grape leaf diseases, where the color features are similar and the lesion distribution is irregular. By optimizing BLS through deep learning network structures, proposing the ABLSS deep learning network model. The model enables fast and accurate recognition and diagnosis of grape leaf diseases, providing support for the development of smart orchard technologies.

Based on the broad learning system, this paper replaces ridge regression with the optimized Adam optimization algorithm and introduces the LTM mechanism to enhance important features and obtain the optimal weight matrix. Meanwhile, the SENet attention mechanism is introduced between the mapping and enhancement layers of broad learning, improving the model’s understanding of image details and global structure. This enhances the model’s ability to recognize small targets and achieves fast and accurate recognition of grape leaf diseases. For precise diagnosis of leaf diseases, an MSCU-Net lesion segmentation model is proposed. The ASPP module effectively captures contextual information at different scales, improving segmentation accuracy while maintaining high resolution. Additionally, the FPN module is introduced to leverage high-resolution detail information and high-level semantic information, increasing the model’s perception of global context and achieving accurate segmentation of edges and small targets. A grape leaf disease grading standard is also formulated.

This paper proposing the ABLSS deep learning network model, it enables fast and accurate recognition and diagnosis of grape leaf diseases. The model gives full play to the advantages of high recognition accuracy of deep learning and fast training speed of broad learning, while avoiding the respective drawbacks of both methods. In the future, we will carry out research on the size and accuracy of the weighting model to further improve the application performance of the model. This research not only allows for accurate and rapid identification of crop diseases in complex natural environments in orchards, providing support for the development of smart orchard technologies;but also holds potential for extension to areas such as damage and strength diagnosis of vulnerable components in industrial machinery.

## Data Availability

The original contributions presented in the study are included in the article/**Supplementary Material**. Further inquiries can be directed to the corresponding authors.
