# Supplementary figures and images for: Deep-broad learning network model for precision identification and diagnosis of grape leaf diseases
Source: Front Plant Sci. 2025 Sep 10;16:1611301. doi: 10.3389/fpls.2025.1611301 (PMC12461735; doi:10.3389/fpls.2025.1611301)

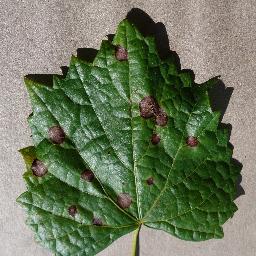

Supplement: Supplementary file 1 [file DataSheet1.zip › B_r00001.JPG]

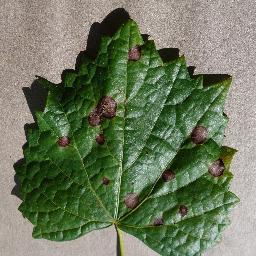

Supplement: Supplementary file 1 [file DataSheet1.zip › B_r00002.JPG]

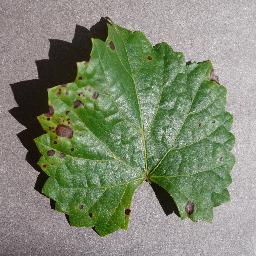

Supplement: Supplementary file 1 [file DataSheet1.zip › B_r00003.JPG]

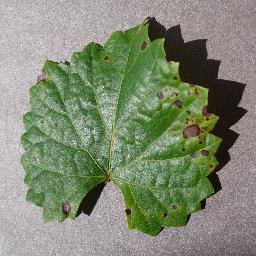

Supplement: Supplementary file 1 [file DataSheet1.zip › B_r00004.JPG]

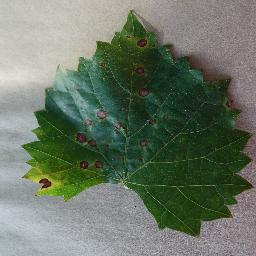

Supplement: Supplementary file 1 [file DataSheet1.zip › B_r00005.JPG]

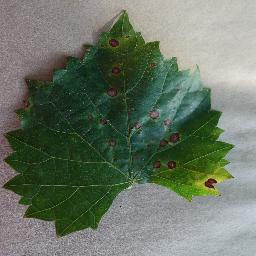

Supplement: Supplementary file 1 [file DataSheet1.zip › B_r00006.JPG]

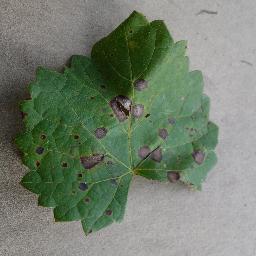

Supplement: Supplementary file 1 [file DataSheet1.zip › B_r00007.JPG]

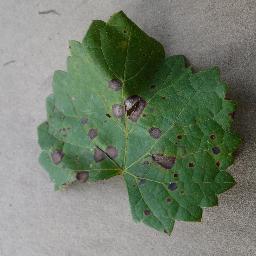

Supplement: Supplementary file 1 [file DataSheet1.zip › B_r00008.JPG]

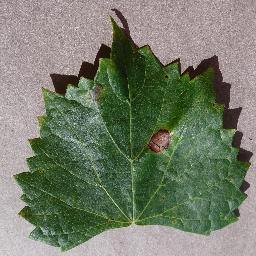

Supplement: Supplementary file 1 [file DataSheet1.zip › B_r00009.JPG]

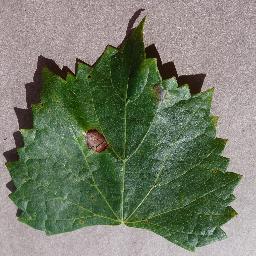

Supplement: Supplementary file 1 [file DataSheet1.zip › B_r00010.JPG]

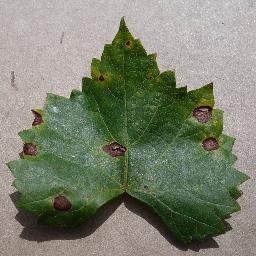

Supplement: Supplementary file 1 [file DataSheet1.zip › B_r00011.JPG]

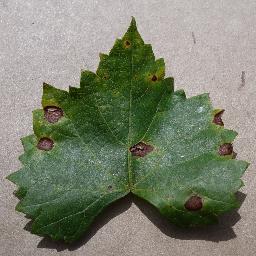

Supplement: Supplementary file 1 [file DataSheet1.zip › B_r00012.JPG]

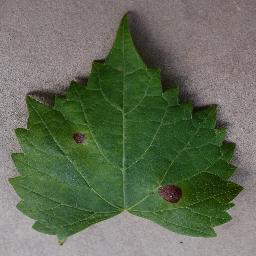

Supplement: Supplementary file 1 [file DataSheet1.zip › B_r00013.JPG]

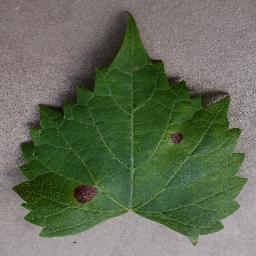

Supplement: Supplementary file 1 [file DataSheet1.zip › B_r00014.JPG]

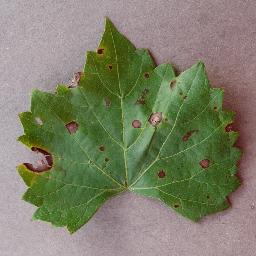

Supplement: Supplementary file 1 [file DataSheet1.zip › B_r00015.JPG]

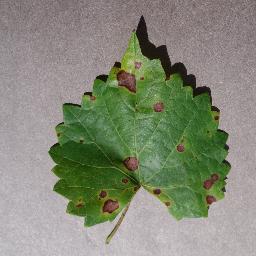

Supplement: Supplementary file 1 [file DataSheet1.zip › B_r00016.JPG]

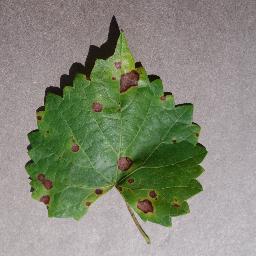

Supplement: Supplementary file 1 [file DataSheet1.zip › B_r00017.JPG]

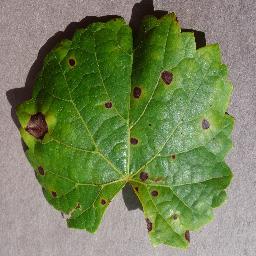

Supplement: Supplementary file 1 [file DataSheet1.zip › B_r00018.JPG]

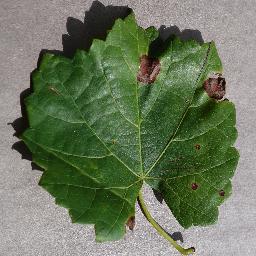

Supplement: Supplementary file 1 [file DataSheet1.zip › B_r00019.JPG]

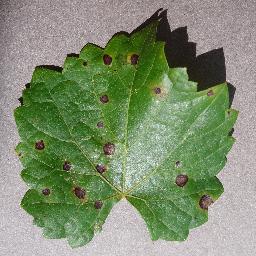

Supplement: Supplementary file 1 [file DataSheet1.zip › B_r00020.JPG]

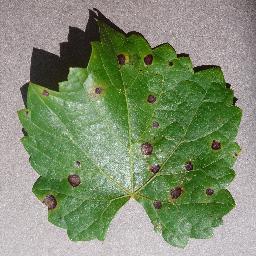

Supplement: Supplementary file 1 [file DataSheet1.zip › B_r00021.JPG]

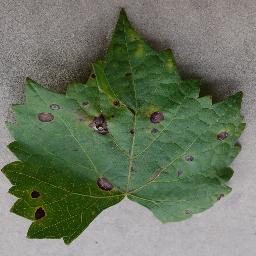

Supplement: Supplementary file 1 [file DataSheet1.zip › B_r00022.JPG]

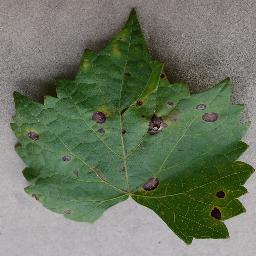

Supplement: Supplementary file 1 [file DataSheet1.zip › B_r00023.JPG]

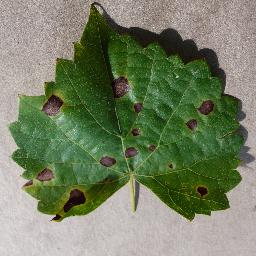

Supplement: Supplementary file 1 [file DataSheet1.zip › B_r00024.JPG]

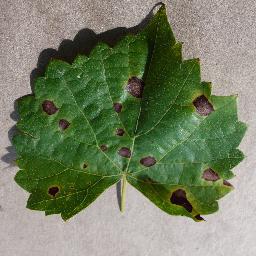

Supplement: Supplementary file 1 [file DataSheet1.zip › B_r00025.JPG]

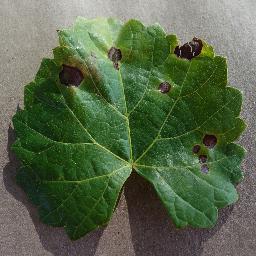

Supplement: Supplementary file 1 [file DataSheet1.zip › B_r00026.JPG]

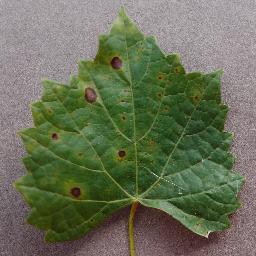

Supplement: Supplementary file 1 [file DataSheet1.zip › B_r00027.JPG]

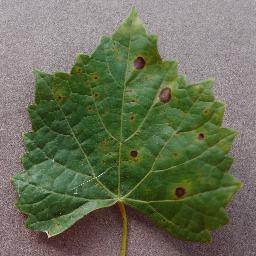

Supplement: Supplementary file 1 [file DataSheet1.zip › B_r00028.JPG]

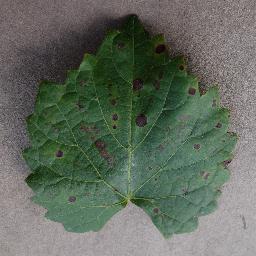

Supplement: Supplementary file 1 [file DataSheet1.zip › B_r00029.JPG]

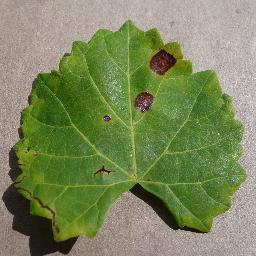

Supplement: Supplementary file 1 [file DataSheet1.zip › B_r00030.JPG]

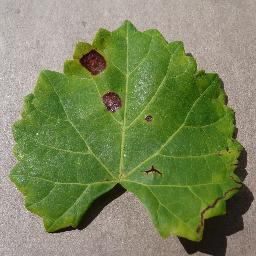

Supplement: Supplementary file 1 [file DataSheet1.zip › B_r00031.JPG]

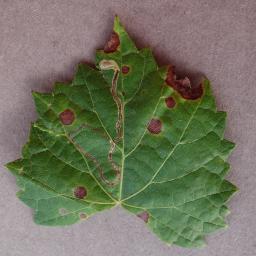

Supplement: Supplementary file 1 [file DataSheet1.zip › B_r00032.JPG]

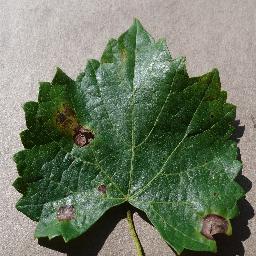

Supplement: Supplementary file 1 [file DataSheet1.zip › B_r00033.JPG]

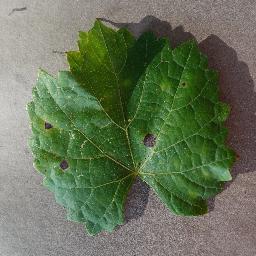

Supplement: Supplementary file 1 [file DataSheet1.zip › B_r00034.JPG]

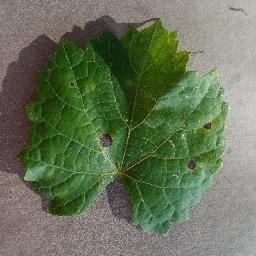

Supplement: Supplementary file 1 [file DataSheet1.zip › B_r00035.JPG]

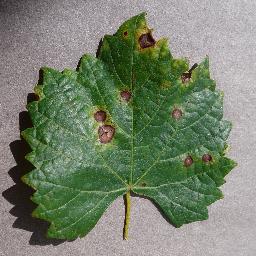

Supplement: Supplementary file 1 [file DataSheet1.zip › B_r00036.JPG]

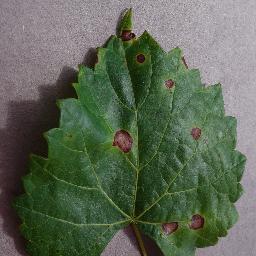

Supplement: Supplementary file 1 [file DataSheet1.zip › B_r00037.JPG]

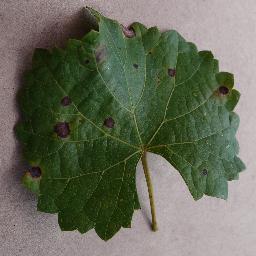

Supplement: Supplementary file 1 [file DataSheet1.zip › B_r00038.JPG]

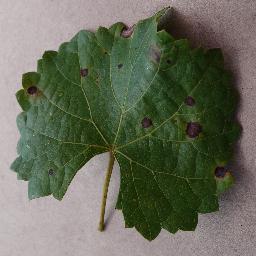

Supplement: Supplementary file 1 [file DataSheet1.zip › B_r00039.JPG]

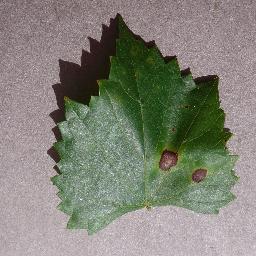

Supplement: Supplementary file 1 [file DataSheet1.zip › B_r00040.JPG]

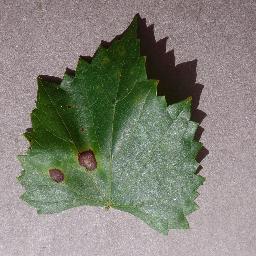

Supplement: Supplementary file 1 [file DataSheet1.zip › B_r00041.JPG]

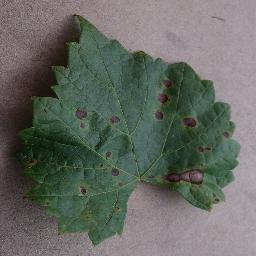

Supplement: Supplementary file 1 [file DataSheet1.zip › B_r00042.JPG]

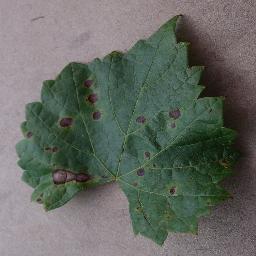

Supplement: Supplementary file 1 [file DataSheet1.zip › B_r00043.JPG]

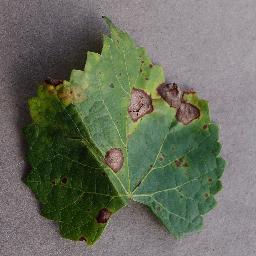

Supplement: Supplementary file 1 [file DataSheet1.zip › B_r00044.JPG]

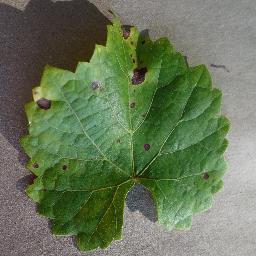

Supplement: Supplementary file 1 [file DataSheet1.zip › B_r00045.JPG]

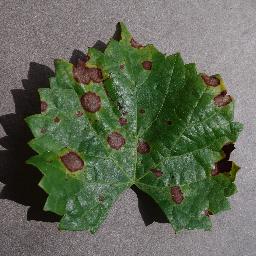

Supplement: Supplementary file 1 [file DataSheet1.zip › B_r00046.JPG]

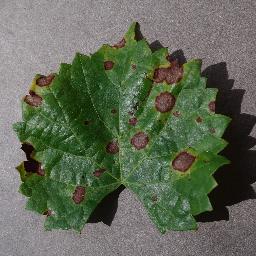

Supplement: Supplementary file 1 [file DataSheet1.zip › B_r00047.JPG]

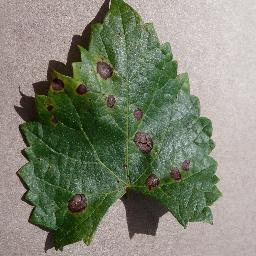

Supplement: Supplementary file 1 [file DataSheet1.zip › B_r00048.JPG]

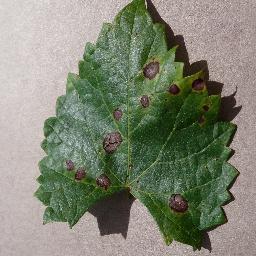

Supplement: Supplementary file 1 [file DataSheet1.zip › B_r00049.JPG]

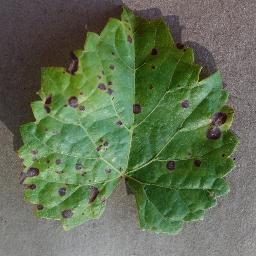

Supplement: Supplementary file 1 [file DataSheet1.zip › B_r00050.JPG]

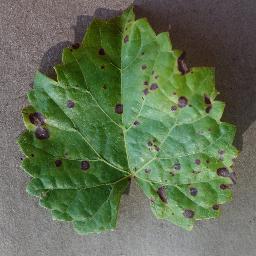

Supplement: Supplementary file 1 [file DataSheet1.zip › B_r00051.JPG]

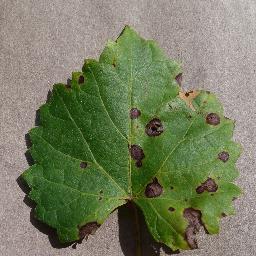

Supplement: Supplementary file 1 [file DataSheet1.zip › B_r00052.JPG]

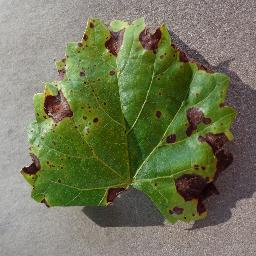

Supplement: Supplementary file 1 [file DataSheet1.zip › B_r00248.JPG]

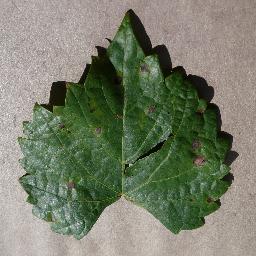

Supplement: Supplementary file 1 [file DataSheet1.zip › B_r00249.JPG]

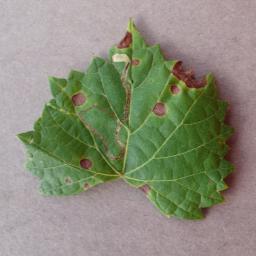

Supplement: Supplementary file 1 [file DataSheet1.zip › B_r00250.JPG]

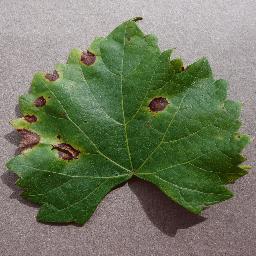

Supplement: Supplementary file 1 [file DataSheet1.zip › B_r00251.JPG]

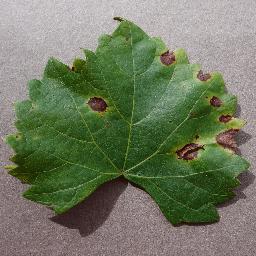

Supplement: Supplementary file 1 [file DataSheet1.zip › B_r00252.JPG]

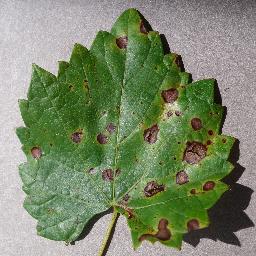

Supplement: Supplementary file 1 [file DataSheet1.zip › B_r00253.JPG]

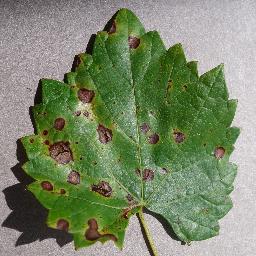

Supplement: Supplementary file 1 [file DataSheet1.zip › B_r00254.JPG]

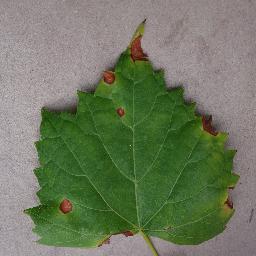

Supplement: Supplementary file 1 [file DataSheet1.zip › B_r00255.JPG]

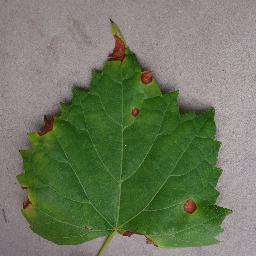

Supplement: Supplementary file 1 [file DataSheet1.zip › B_r00256.JPG]

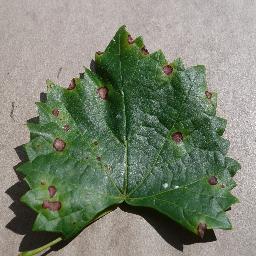

Supplement: Supplementary file 1 [file DataSheet1.zip › B_r00257.JPG]

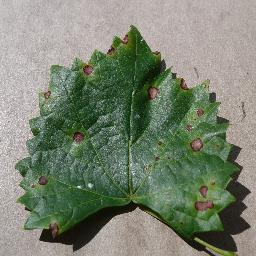

Supplement: Supplementary file 1 [file DataSheet1.zip › B_r00258.JPG]

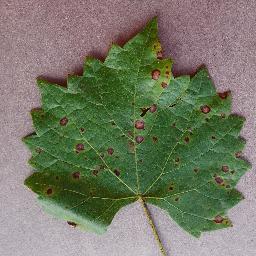

Supplement: Supplementary file 1 [file DataSheet1.zip › B_r00259.JPG]

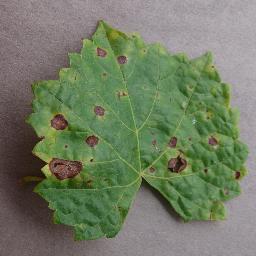

Supplement: Supplementary file 1 [file DataSheet1.zip › B_r00260.JPG]

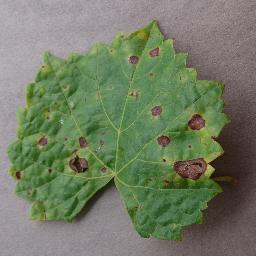

Supplement: Supplementary file 1 [file DataSheet1.zip › B_r00261.JPG]

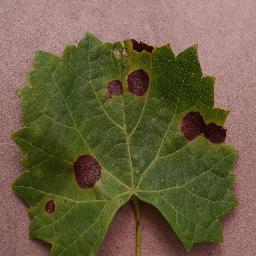

Supplement: Supplementary file 1 [file DataSheet1.zip › B_r00262.JPG]

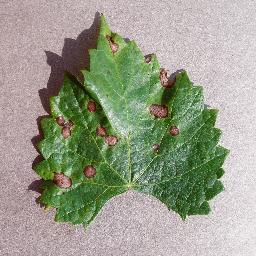

Supplement: Supplementary file 1 [file DataSheet1.zip › B_r00263.JPG]

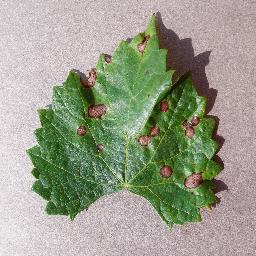

Supplement: Supplementary file 1 [file DataSheet1.zip › B_r00264.JPG]

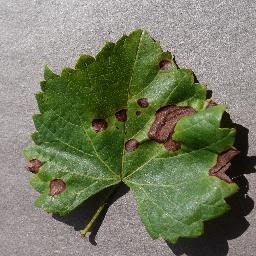

Supplement: Supplementary file 1 [file DataSheet1.zip › B_r00265.JPG]

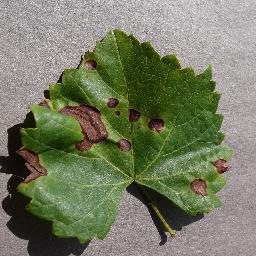

Supplement: Supplementary file 1 [file DataSheet1.zip › B_r00266.JPG]

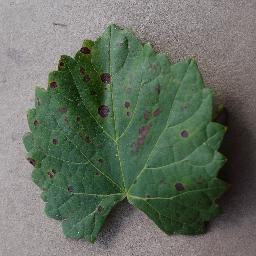

Supplement: Supplementary file 1 [file DataSheet1.zip › B_r00267.JPG]

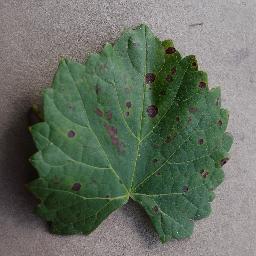

Supplement: Supplementary file 1 [file DataSheet1.zip › B_r00268.JPG]

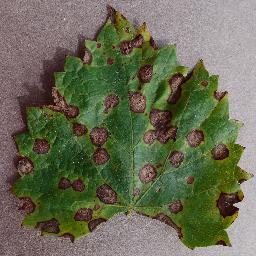

Supplement: Supplementary file 1 [file DataSheet1.zip › B_r00269.JPG]

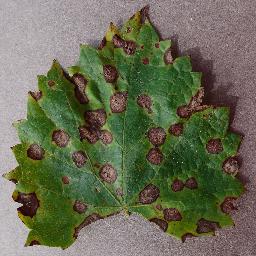

Supplement: Supplementary file 1 [file DataSheet1.zip › B_r00270.JPG]

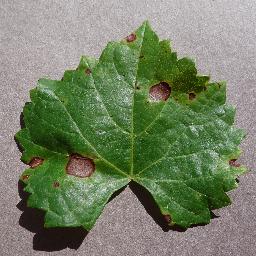

Supplement: Supplementary file 1 [file DataSheet1.zip › B_r00271.JPG]

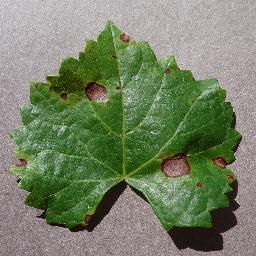

Supplement: Supplementary file 1 [file DataSheet1.zip › B_r00272.JPG]

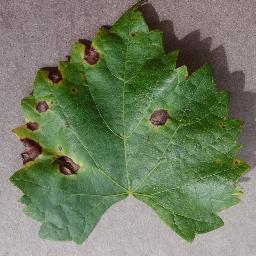

Supplement: Supplementary file 1 [file DataSheet1.zip › B_r00273.JPG]

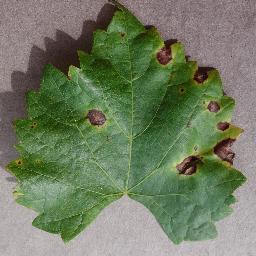

Supplement: Supplementary file 1 [file DataSheet1.zip › B_r00274.JPG]

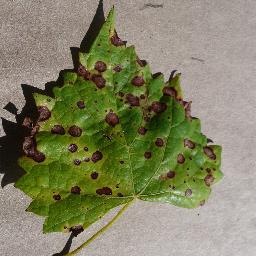

Supplement: Supplementary file 1 [file DataSheet1.zip › B_r00275.JPG]

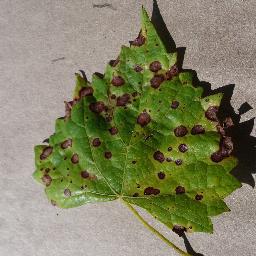

Supplement: Supplementary file 1 [file DataSheet1.zip › B_r00276.JPG]

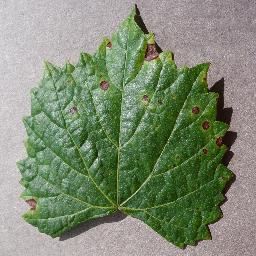

Supplement: Supplementary file 1 [file DataSheet1.zip › B_r00277.JPG]

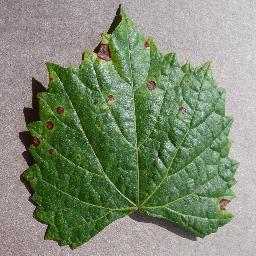

Supplement: Supplementary file 1 [file DataSheet1.zip › B_r00278.JPG]

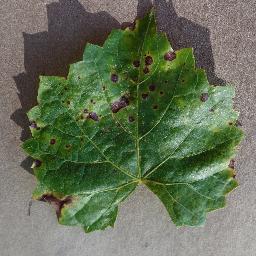

Supplement: Supplementary file 1 [file DataSheet1.zip › B_r00279.JPG]

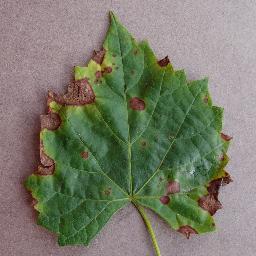

Supplement: Supplementary file 1 [file DataSheet1.zip › B_r00280.JPG]

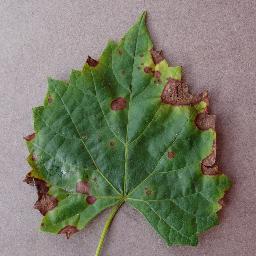

Supplement: Supplementary file 1 [file DataSheet1.zip › B_r00281.JPG]

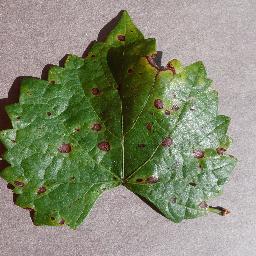

Supplement: Supplementary file 1 [file DataSheet1.zip › B_r00282.JPG]

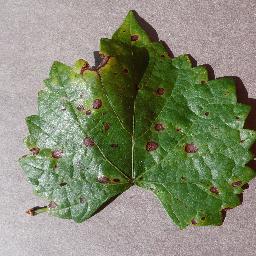

Supplement: Supplementary file 1 [file DataSheet1.zip › B_r00283.JPG]

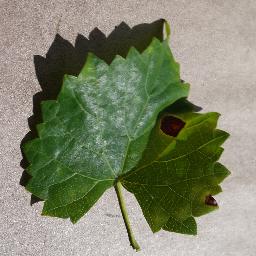

Supplement: Supplementary file 1 [file DataSheet1.zip › B_r00284.JPG]

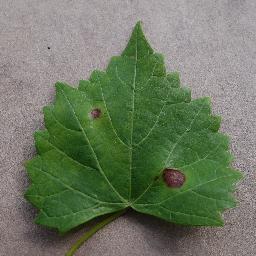

Supplement: Supplementary file 1 [file DataSheet1.zip › B_r00285.JPG]

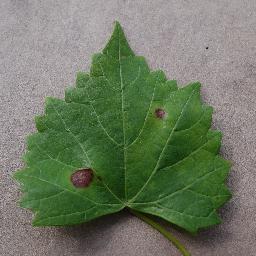

Supplement: Supplementary file 1 [file DataSheet1.zip › B_r00286.JPG]

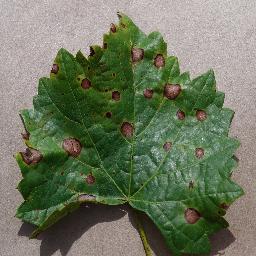

Supplement: Supplementary file 1 [file DataSheet1.zip › B_r00287.JPG]

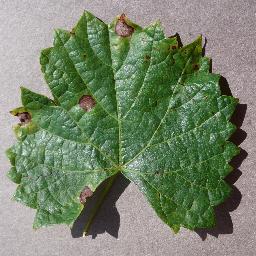

Supplement: Supplementary file 1 [file DataSheet1.zip › B_r00288.JPG]

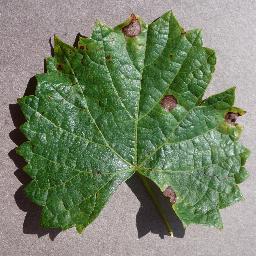

Supplement: Supplementary file 1 [file DataSheet1.zip › B_r00289.JPG]

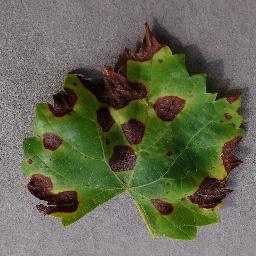

Supplement: Supplementary file 1 [file DataSheet1.zip › B_r00290.JPG]

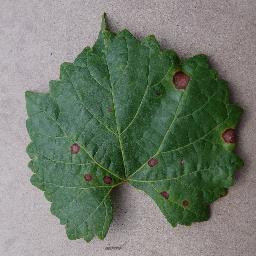

Supplement: Supplementary file 1 [file DataSheet1.zip › B_r00291.JPG]

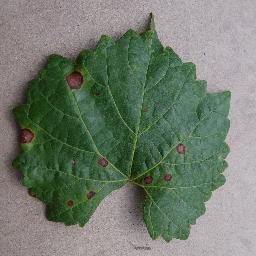

Supplement: Supplementary file 1 [file DataSheet1.zip › B_r00292.JPG]

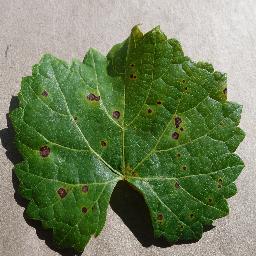

Supplement: Supplementary file 1 [file DataSheet1.zip › B_r00293.JPG]

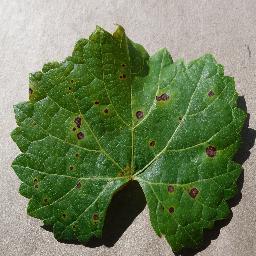

Supplement: Supplementary file 1 [file DataSheet1.zip › B_r00294.JPG]

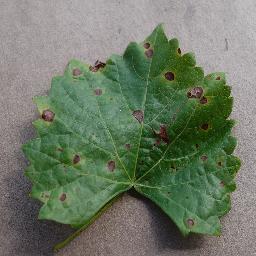

Supplement: Supplementary file 1 [file DataSheet1.zip › B_r00295.JPG]
